# Mapping Cell Types and Efferent Pathways in the Ascending Relaxin-3 System of the Nucleus Incertus

**DOI:** 10.1523/ENEURO.0272-20.2020

**Published:** 2020-11-04

**Authors:** Nailyam Nasirova, Lely A. Quina, Glenn Morton, Andrew Walker, Eric E. Turner

**Affiliations:** 1 Center for Integrative Brain Research, Seattle Children’s Research Institute, Seattle, WA 98101; 2 Department of Psychiatry and Behavioral Sciences, University of Washington, Seattle, WA 98195

**Keywords:** GABA, neuromedin-b, pons, relaxin-3, tegmentum, transgenic model

## Abstract

Relaxin-3 (Rln3) is an insulin-family peptide neurotransmitter expressed primarily in neurons of the nucleus incertus (NI) of the pontine tegmentum, with smaller populations located in the deep mesencephalon (DpMe) and periaqueductal gray (PAG). Here, we have used targeted recombination at the *Rln3* gene locus to generate an *Rln3^Cre^* transgenic mouse line, and characterize the molecular identity and axonal projections of Rln3-expressing neurons. Expression of Cre recombinase in *Rln3^Cre^* mice, and the expression of Cre-mediated reporters, accurately reflect the expression of Rln3 mRNA in all brain regions. In the NI, Rln3 mRNA is expressed in a subset of a larger population of tegmental neurons that express the neuropeptide neuromedin-b (NMB). These Rln3-expressing and NMB-expressing neurons also express the GABAergic marker GAD2 but not the glutamatergic marker Slc17a6 (VGluT2). Cre-mediated anterograde tracing with adeno-associated viruses (AAVs) shows that the efferents of the Rln3-expressing neurons in the DpMe and PAG are largely confined to the brain regions in which they originate, while the NI-Rln3 neurons form an extensive ascending system innervating the limbic cortex, septum, hippocampus, and hypothalamus. Viral anterograde tracing also reveals the potential synaptic targets of NI-Rln3 neurons in several brain regions, and the distinct projections of Rln3-expressing and non-expressing neurons in the pontine tegmentum. Rabies virus (RV)-mediated transsynaptic retrograde tracing demonstrates a probable synaptic link between NI-Rln3 neurons and GABAergic neurons in the septum, with implications for the modulation of neural activity in the septo-hippocampal system. Together, these results form the basis for functional studies of the NI-Rln3 system.

## Significance Statement

Relaxin-3 (Rln3) is a peptide neurotransmitter expressed mainly in the nucleus incertus (NI) of the pons. Rln3 neurons project to the limbic cortex, septum, hippocampus, and hypothalamus, in a way that resembles the ascending brain systems expressing dopamine, serotonin, and norepinephrine. Roles in arousal and stress responses have been proposed for NI-Rln3 neurons, but their function is not well understood. We have defined the molecular signature of NI-Rln3 neurons in terms of the expression markers for GABA, the principal inhibitory neurotransmitter of the CNS, and neuromedin-b (NMB), another neuropeptide expressed in the pons. A transgenic mouse model for the Rln3 system has allowed the projections of the Rln3 neurons to be mapped in detail, and will facilitate functional studies of this pathway.

## Introduction

Relaxin-3 (Rln3) is an insulin-family peptide neurotransmitter with very restricted expression in the CNS. Rln3-expressing cell bodies are found primarily in the nucleus incertus (NI), with smaller populations located in the deep mesencephalon (DpMe) and periaqueductal gray (PAG). In contrast, Rln3-containing fibers and the principal Rln3 receptor, Rxfp3, are widely distributed in the rodent (Ma et al., 2007; Smith et al., 2010) and primate (Ma et al., 2009a) forebrain and diencephalon. The location of the Rln3-expressing neurons that give rise to this distributed system has not been precisely defined, but anatomic projections from the area of the NI are known to innervate wide brain regions (Goto et al., 2001; Olucha-Bordonau et al., 2003). Thus, the NI-Rln3 system resembles the dopamine, serotonin, and noradrenergic systems in that it consists of a tegmental nucleus that sends ascending projections to multiple regions important to behavioral regulation, including the limbic cortex, septum, hippocampus, and hypothalamus.

Consistent with the wide distribution of the Rln3 projections, several roles have been proposed for this system in the regulation of behavior. Mice with null mutations in Rxfp3 are viable and fertile, but have a number of subtle behavioral deficits including reduced running wheel activity (Hosken et al., 2015), and changes in operant sucrose seeking (Walker et al., 2015a) and stress-related alcohol consumption (Walker et al., 2015b). Rln3 null mice are also viable, but show subtle changes in anxiety measures (Watanabe et al., 2011). Based on these genetic models, and also on pharmacological studies using Rln3 agonists and antagonists, Rln3/Rxfp3 have been postulated to modulate arousal and stress responses (Smith et al., 2014; Ma and Gundlach, 2015). However, genetic and pharmacological manipulations of Rln3 and its receptor Rxfp3 system only partly address the function of this neural pathway, because the principal neurotransmitter of the NI-Rln3 system is GABA, which is co-expressed with the Rln3 peptide (Ma et al., 2007).

Much of the prior work on the function of the NI has focused on its role in the regulation of hippocampal theta rhythm, an electroencephalogram (EEG) pattern characterized by coherent oscillations of local field potential and phase-locked neuronal firing in the 4- to 10-Hz frequency band observable during active awake behavior (Pignatelli et al., 2012). These oscillations have been described as being related to sensorimotor integration, spatial navigation, and memory (Buzsáki and Moser, 2013; Hasselmo and Stern, 2014). Theta activity is observed in multiple brain regions receiving Rln3 fibers, including CA1 and CA3 of the hippocampus, the medial septum (MS), and the retrosplenial cortex (RS; Ma et al., 2007; Smith et al., 2010). The function of the NI in modulation of theta activity has recently been explored using transgenic mouse models. One transgenic system allowed the manipulation of all GABAergic neurons in the area of the NI (Szőnyi et al., 2019), and another targeted a specific population of neurons in the region that express the neuropeptide neuromedin-b (NMB; Lu et al., 2020). These studies reported inhibition and enhancement of hippocampal theta activity, respectively, with the stimulation of these cell populations. However, the identity of the neurons examined in these studies with respect to the ascending NI-Rln3 system has not been completely defined.

Here, we have used targeted recombination at the *Rln3* gene locus in embryonic stem cells (ESCs) to generate an *Rln3^Cre^* transgenic mouse line. Crosses of *Rln3^Cre^* mice with genetic reporter mouse strains faithfully reproduced the expression of Rln3 mRNA in the mesencephalon and NI. The NI neurons labeled by *Rln3^Cre^* are GABAergic, and are a subset of a larger group of NMB-expressing neurons in the pontine tegmentum. Using *Rln3^Cre^*-driven expression of adeno-associated viruses (AAVs), we performed anterograde tracing of the distinct projections of the Rln3-expressing neurons in the NI, DpMe, and PAG. The NI-Rln3 neurons also project to distinct target areas compared with their Rln3-negative neighbors in the pons. Consistent with a role in regulating theta activity in the septohippocampal system, NI-Rln3 neurons make synaptic connections with GABAergic neurons in the septum. The *Rln3^Cre^* transgenic mouse strain will provide an important new tool for specific functional studies of the Rln3-expressing brain systems.

## Materials and Methods

### Targeting the *Rln3* gene locus in ESCs and generation of *Rln3^Cre^* mice

*Rln3^Cre^* mice were generated by gene targeting in G4 129S6B6F1 hybrid mouse ESCs (George et al., 2007). ESCs were electroporated with a gene targeting construct encoding an IRES, GFP-Cre fusion protein, and PGK-neomycin expression cassette. The insertion was targeted to the 3′-untranslated region of the *Rln3* gene, encoded on the (−) strand of mouse Chr8, immediately downstream of the Rln3 stop codon at Chr8:84 043,093–84 043,095 (NCBI mouse genome GRCm38/mm10). The short arm 5′ to the insertion site consisted of 2037 nucleotides spanning Chr8: 84,043,073–840,45,109, and the long arm 3′ to the insertion site consisted of 4109 nucleotides from Chr8:84,038,958–84,043,066. NEO-resistant ESC clones were screened first by polymerase chain reaction using oligonucleotides spanning the 5′ (short)-homology arm, then by Southern hybridization using a probe within the 3′ (long) homology arm. ESCs with the correctly targeted gene structure were used to generate founder animals by blastocyst injection. In order to excise the Neomycin selection cassette, founder mice were interbred with the strain Gt(ROSA)26Sor^tm1(FLP1)Dym^/J (“FLPeR” mice, Jax #003946) and the absence of the NEO coding sequence was confirmed in the F1 by PCR. NEO-excised mice on a mixed genetic background were subsequently bred to C57BL/6NCrl (Charles River) and maintained on this genetic background.

### Other mouse strains

Other mouse strains included the Cre-dependent tdTomato reporter strain *Ai14* (*Gt(ROSA)26Sor^tm14(CAG-tdTomato)Hze/J^*; Madisen et al., 2010; Jax #007914), the Cre-dependent ZsGreen reporter strain *Ai6* (*Gt(ROSA)26Sor^tm6(CAG-ZsGreen1)Hze/J^*; Madisen et al., 2010; Jax #007906), and *Gad2^IRES-Cre^* (*Gad2^tm2(cre)Zjh/J^*; Taniguchi et al., 2011; Jax #010802). All strains were maintained on a C57BL/6NCrl genetic background. Both male and female mice were used for anatomic and gene expression studies.

### Immunofluorescence and *in situ* hybridization

Mouse brain tissue was prepared by fixation via transcardial perfusion with 4% paraformaldehyde. Brains were then removed and equilibrated in graded sucrose solutions, frozen at −80°C in OCT solution, and cryosectioned at 25 μm for fluorescence/immunofluorescence imaging. Tissue processed in this way was suitable for imaging of endogenous protein fluorescence, immunofluorescence, and fluorescence *in situ* hybridization (FISH). Primary antiserum used included rabbit anti-tyrosine hydroxylase (AB152, EMD Millipore, RRID:AB_390204). The endogenous fluorescence of the tdTomato reporter was enhanced with rabbit anti-red fluorescent protein (600-401-379, Rockland Immunochemicals, RRID:AB_2209751). Immunofluorescence for Rln3 peptide was performed in the laboratory of Andrew Gundlach, Florey Institute, Melbourne, using a mouse monoclonal antibody (mAb), and data were provided as unpublished results. The development of the hybridoma HK4-144-10 producing a mAb recognizing a conserved N-terminal Rln3 peptide (Kizawa et al., 2003), the use of this mAb for immunostaining in the NI of the rat, including blocking of the signal by the immunizing peptide (Tanaka et al., 2005), and the validation of the specificity of the mAb in Rln3 knock-out mice (Watanabe et al., 2011), have been previously published. Multi-channel FISH was performed with the RNAscope Multiplex Fluorescent V2 kit, according to the manufacturer’s instructions (Advanced Cell Diagnostics). The probes used included: Cre recombinase, #312281-C2 (channel 2); EGFP, #400281-C2; Mm-Gad2, #439371-C2; Mm-Nmb, #459931-C3 (channel 3); Mm-Rln3 (channel 1), #459921; Mm-Slc17a6 (channel 1), #319171 (VGluT2).

### Anterograde and retrograde tracing: general methods

The targeted coordinates for each anterograde or retrograde tracing injection, based on a standard atlas (Paxinos and Franklin, 2001), appear in the figure legends. The detailed methods used here for anterograde tract tracing with iontophoretic injection of AAV have been published in conjunction with the Allen Mouse Brain Connectivity Atlas (Harris et al., 2012; Oh et al., 2014). Animals were fixed by transcardial perfusion with 4% paraformaldehyde at 14–21 d after injection and processed as described above.

### Anterograde tract tracing: viruses

Anterograde tracing was performed using AAV, including Cre-activated (Flex) and Cre-silenced (Fas) vectors (“Cre-on, Cre-off” system). Viral stocks were prepared at the University of Pennsylvania Gene Therapy Program Vector Core (https://gtp.med.upenn.edu/core-laboratories-public/vector-core). All viruses used were AAV capsid strain 1. For Cre-dependent labeling of cell bodies and axons we used AAV pCAG.FLEX.tdTomato.WPRE (“FLEX-tdT,” Addgene plasmid #51503) or AAV pCAG.FLEX.EGFP.WPRE (“FLEX-GFP,” Addgene plasmid #51502). Enhanced labeling of presynaptic areas was performed by Cre-dependent viral expression of a synaptophysin-EGFP fusion protein (sypGFP). The plasmid pCAG.Flex.sypEGFP.WPRE (“FLEX-sypGFP”) was constructed by replacing the EGFP moiety of pCAG-FLEX-EGFP-WPRE with the sypEGFP construct from phSyn1(S)-FLEX-tdTomato-T2A-SypEGFP-WPRE (Addgene #51509) by Julie Harris, Karla Hirokawa, and Hong Gu of the Allen Institute for Brain Science (gift of Julie Harris). In most experiments the tdTomato axonal tracer and the sypGFP synaptic tracer viruses were co-injected. Cre-inactivated expression was performed with pAAV-Ef1a-FAS-tdTomato-WPRE (“FAS-tdT,” Addgene #37092; Saunders et al., 2012), which was co-injected with the FLEX-GFP or FLEX-sypGFP virus.

### Transsynaptic tracing

For monosynaptic retrograde tracing, the helper virus AAV1-Syn-DIO-TVA66T-dTom-CVS N2cG, (AAV1-N2cG) a tricistronic virus which expresses the pseudotyping receptor TVA, tdTomato, and the rabies glycoprotein G (Lo et al., 2019) was injected by pressure injection into the MS of *Gad2^Cre^* mice, followed by the rabies virus (RV) EnvA CVS-N2cΔG-histone-eGFP (RV-GFP), injected 21 d later into the same location. The injection coordinates were: AP: 0.74, ML: 0.00, DV: 4.2, and the injected volume was 200 nl. AAV1 and RV for transsynaptic tracing were the gifts of Shenqin Yao and Ali Cetin (Allen Institute for Brain Science). Further details regarding rabies reagents are available on request from Dr. Yao. Mice were euthanized 10 d later and the brains were processed as described above to visualize the nuclear GFP signal or virally expressed GFP mRNA in presynaptic neurons in the pontine tegmentum.

## Results

### Generation of an *Rln3^Cre^* transgenic mouse strain

**Figure 1.**
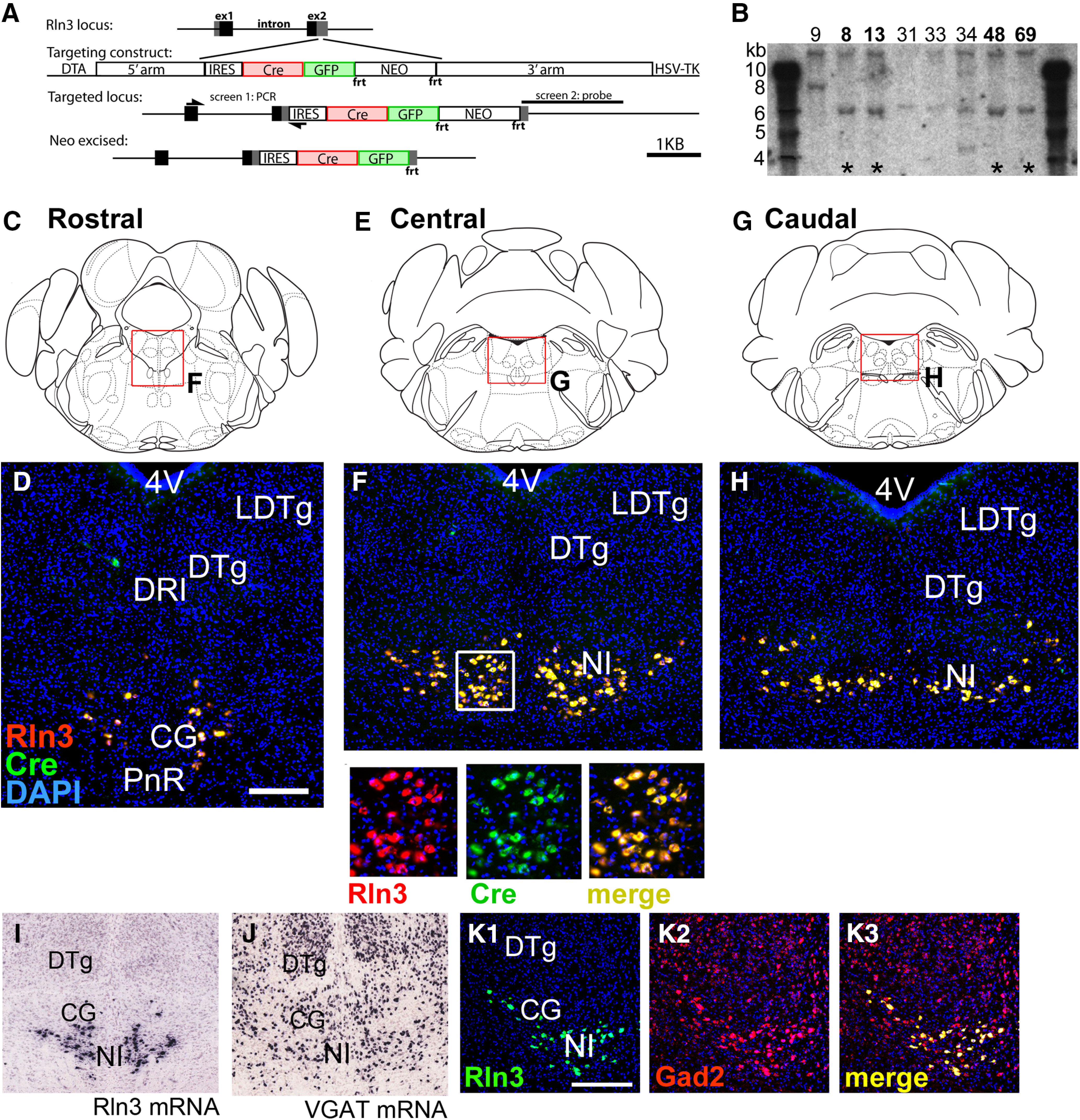
Generation and characterization of an Rln3-Cre transgenic line. ***A***, Targeting of the *Rln3* gene locus. A transgenic construct encoding a Cre-GFP fusion protein and a neomycin (NEO) resistance marker was prepared targeting to the 3′-untranslated part of exon 2 of the *Rln3* gene, and electroporated into ESCs (Materials and Methods). ESC clones were selected for neomycin resistance and were initially screened by PCR across the 5′-flanking arm, then by Southern hybridization using a 1.9-kb probe to the 3′-flanking arm. ***B***, Autoradiogram of a Southern blot of an XbaI genomic digest detecting a >10-kb band in the native *Rln3* locus and a novel 6-kb band in the targeted locus. Asterisks indicate ESC lines with correct targeting. Clone 9 has a randomly integrated transgene, and was negative in the initial PCR screen for correct targeting. Clones 8,13, 48, and 69 have the digestion pattern expected for a single correct integrant. ***C–H***, FISH showing co-expression of mRNA for Cre and Rln3 in the rostral (***C***, ***D***), central (***E***, ***F***), and caudal (***G***, ***H***) nucleus incertus of a heterozygous *Rln3^Cre^* mouse. Insets in ***F*** show complete concordance of Cre and Rln3 expression. ***I***, ***J***, Expression of Rln3 (***L***) and VGAT (***M***) mRNA in the NI and surrounding CG; ISH data are derived from the Allen Brain Atlas (Ng et al., 2009), case numbers 73929581 and 72081554, respectively. ***K***, Confocal images of FISH using probes for Rln3 and VGAT mRNA, at a rostrocaudal level similar to that shown in ***E***, ***F***. ***K1***, Rln3 signal, ***K2***, Gad2 signal, ***K3***, merged signals. The left NI is shown. Rln3 expression is restricted to the NI, while VGAT is widely expressed in the surrounding CG. All of the NI-Rln3 neurons in the field co-express VGAT. 4V, fourth ventricle; CG, central pontine gray; DRI, dorsal raphe, interfascicular part; DTg, dorsal tegmental nucleus; LDTg, laterodorsal tegmental nucleus; NI, nucleus incertus; PnR, pontine raphe nucleus. Scale bar: 200 μm (***D***, ***K***).

*Rln3^Cre^* transgenic mice were generated by gene targeting in ESCs. A targeting construct encoding an IRES, a GFP/Cre fusion protein, and a neomycin (NEO) resistance cassette (NEO) was targeted to 3′-untranslated region of the *Rln3* locus, in a location immediately downstream from the stop codon in the *Rln3* open reading frame (Fig. 1*A*; Materials and Methods). Neomycin resistant ESC clones were screened first by PCR across the 5′-homology arm of the targeting construct, then for correct integration by Southern blotting using a probe to the 3′-homology arm (Fig. 1*B*). Two ESC clones were selected for blastocyst injection to generate founder lines. For Flp-recombinase mediated excision of the NEO cassette, the highly chimeric male founders from one founder line were interbred with female mice bearing the Flp-deleter allele Gt(ROSA)26Sor^tm1(FLP1)Dym^/J (“FLPeR” mice, Jax #003946) and the absence of the NEO coding sequence was confirmed in the F1 generation by PCR. NEO-excised mice on a mixed genetic background were subsequently bred to C57BL/6 (Charles River) and maintained on this genetic background.

To test the fidelity of Cre expression in *Rln3^Cre^* mice, we first compared Cre mRNA expression to endogenous Rln3 mRNA expression using FISH (RNAscope; Fig. 1*C–H*). An exact correspondence was observed between Cre and Rln3 expression in the NI at all rostrocaudal levels, indicating the Rln3-IRES-Cre/GFP transgene is efficiently and specifically transcribed. However, examination of Rln3 peptide immunoreactivity, using a well characterized mAb (Kizawa et al., 2003; Watanabe et al., 2011; Haidar et al., 2017), revealed decreased expression in the NI of *Rln3^Cre/+^* mice, and very low (or negligible) signal in *Rln3^Cre/Cre^* mice, indicating that the *Rln3^Cre^* allele is hypomorphic (A. L. Gundlach and S. Ma, The Florey Institute of Neuroscience and Mental Health, Melbourne, Australia; unpublished data). We interpret the attenuated Rln3 immunoreactivity to mean that, for an unknown reason, the Rln3 peptide is not efficiently translated from the dicistronic Rln3-IRES-Cre mRNA. All experiments reported here were performed with *Rln3^Cre/+^* mice.

NI-Rln3 neurons lie within the central gray of the pons (CG), where they comprise a relatively small fraction of the total cell population, as can be seen using gene expression data from a public database (Fig. 1*I*; Ng et al., 2009). Although NI-Rln3 neurons have been reported to be GABAergic (Ma et al., 2007), many of the surrounding cells also express the GABA transporter VGAT (Fig. 1*I*,*J*), so the GABAergic phenotype does not distinguish NI-Rln3 neurons from their neighbors. In order to confirm the GABAergic phenotype of NI-Rln3 neurons, we performed FISH using probes for Rln3 and GAD2, a GABA biosynthetic enzyme (Fig. 1*K*). GAD2 mRNA expression was widespread in the CG, including, as expected, all of the Rln3-expressing neurons. These results show why it is not possible to specifically target the NI for functional experiments, such as optogenetic activation or silencing, using a transgenic driver targeting a widely-expressed marker like *Gad2^Cre^*, or by injection into animals without any genetic targeting system.

**Figure 2.**
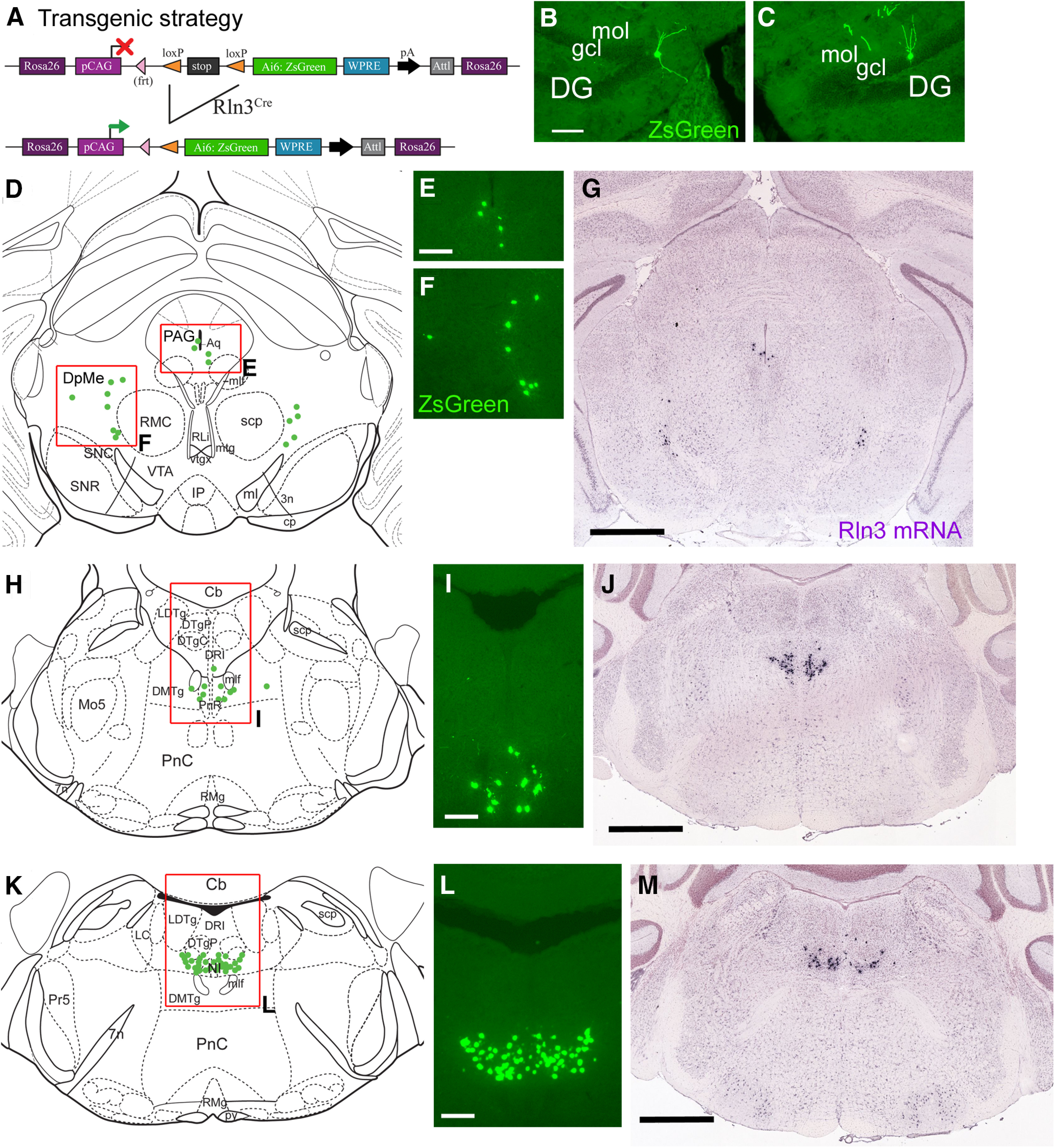
Fidelity of *Rln3^Cre^*-driven genetic reporter expression. ***A***, Transgenic reporter strategy: *Rln3^Cre^* mice were interbred with the genetic reporter strain *Ai6*, which conditionally expresses a ZsGreen reporter from the *Gt(Rosa)26Sor* locus. At each brain level, induced expression of the reporter was compared with endogenous expression of Rln3 mRNA in the Allen Brain Atlas (Allen Atlas case 73929581). ***B***, ***C***, Isolated examples of rare neurons showing ectopic reporter expression in the hippocampus. ***D–G***, Expression in the midbrain at a level corresponding to bregma −3.5 in a standard atlas (Paxinos and Franklin, 2001). Reporter expression appears in the midbrain PAG (***E***) and in an area dorsal to the substantia nigra (***F***). ***H–J***, Expression in the pontine raphe/rostral nucleus incertus, bregma −5.2. The view in ***J*** is slightly caudal to the views in ***H***, ***I*** and includes more neurons dorsal to the mlf. ***K–M***, Expression in the central part of the nucleus incertus, bregma −5.40. DG, dentate gyrus; gcl, granular cell layer; mol, molecular layer of the dentate gyrus. Scale bars: 100 μm (***B***), 200 μm (***E***, ***I***, ***L***), and 1 mm (***G***, ***J***, ***M***).

In order to test Cre-mediated gene expression throughout the entire neural axis of *Rln3^Cre^* mice, we interbred this strain with a genetic reporter line *Ai6*, which exhibits Cre-dependent expression of the fluorescent reporter ZsGreen (Fig. 2*A*). *Rln3^Cre^/Ai6* brain sections were aligned with Rln3 mRNA expression images from the Allen Brain Atlas (Ng et al., 2009). Examination of the entire CNS rarely revealed ZsGreen-labeled neurons outside of the brain regions previously described as containing Rln3 neurons (Smith et al., 2010). The only exception noted was very sporadic expression in the dentate gyrus (DG; Fig. 2*B*,*C*). Otherwise, ZsGreen expression strongly resembled that of endogenous message Rln3 mRNA, including the DpMe and PAG of the mesencephalon (Fig. 2*D–G*), the rostral part of the NI cell group residing in the pontine raphe (Fig. 2*H–J*), and in the central part of the NI (Fig. 2*K–M*). This result is important not only because it demonstrates the spatial fidelity of Cre expression but also because it excludes other areas of *Rln3^Cre^* expression in the developing brain, since recombination of the reporter locus during development would lead to persistent expression in the adult.

**Figure 3.**
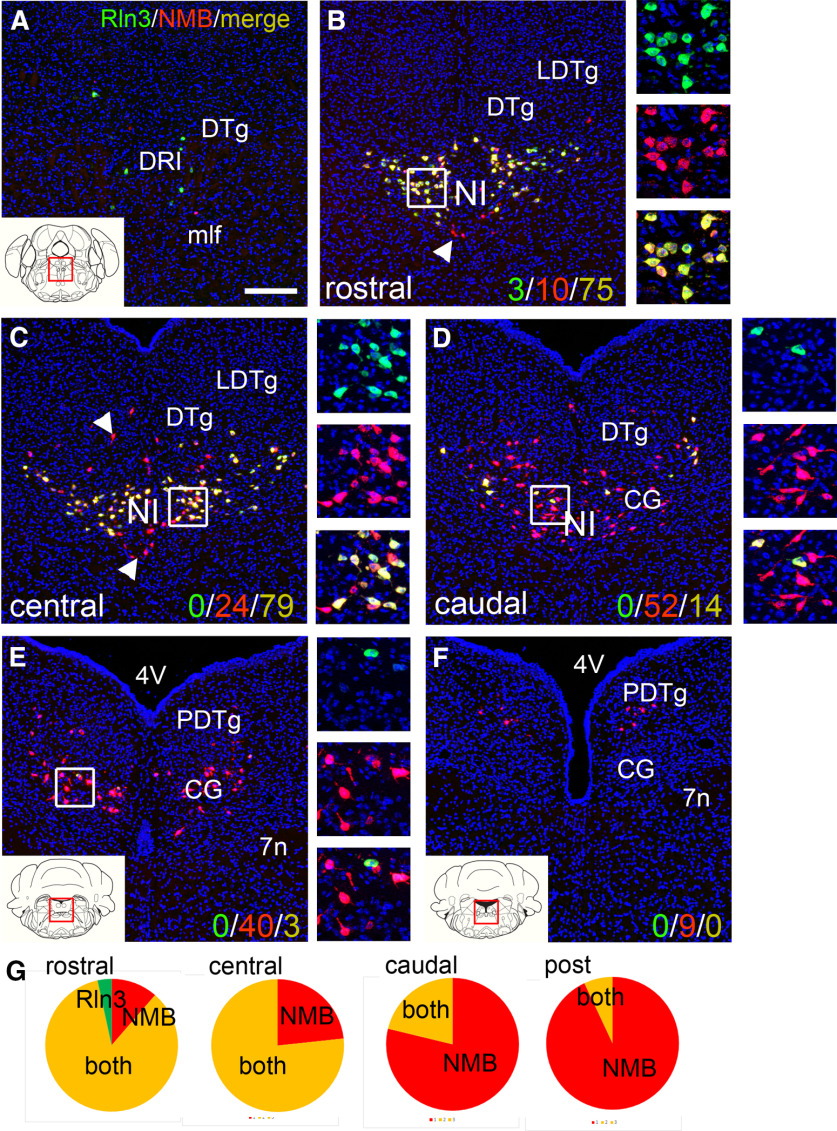
Expression and co-expression of Rln3 and NMB in the pontine central gray. ***A–F***, FISH was used to examine Rln3 and NMB mRNA expression in six equally spaced sections at levels corresponding to bregma −5.0 to bregma −5.8 in a standard atlas (Paxinos and Franklin, 2001). Z-stacks of confocal images of the cells in the boxed area of each panel are shown at right. ***A***, Area rostral to the NI at bregma −5.0. Rostral, central, and caudal in ***B–D*** represent NI levels approximately equivalent to the images in Figure 1 but are shifted slightly caudally relative to that figure. ***B***, Corresponds to bregma −5.16 relative to a standard atlas, (***C***) at bregma −5.32, and (***D***) at bregma −5.48. Sections ***E***, ***F*** lie caudal to the area designated NI in a standard atlas, (***E***) at bregma −5.64, and (***F***) at bregma −5.80. The numerical values in ***B–F*** in the lower right of the panels represent cell counts for neurons expressing Rln3 alone (red), NMB alone (green), and both markers (yellow). In the rostral and central NI, neurons expressing NMB alone were identified dorsal and ventral to the NI-Rln3 neurons, near the midline (***B***, ***C***, arrows); in the caudal NI cells expressing NMB alone predominate (***D***). The area posterior to the NI has a large population of cells which express NMB alone (***E***, ***F***). ***G***, Graphical representation of the distribution of Rln3 and NMB expression in images (***B–E***). 4V, fourth ventricle; 7n, seventh nerve; CG, central pontine gray; DRI, dorsal raphe, interfascicular part; DTg, dorsal tegmental nucleus; LDTg, laterodorsal tegmental nucleus; mlf, medial longitudinal fasciculus; NI, nucleus incertus; PDTg, posterodorsal tegmental nucleus. Scale bar: 200 μm (***A***).

A subset of neurons in the pontine tegmentum express the neuropeptide NMB, and *NMB^Cre^* mice have been used to target the NI in tract-tracing and optogenetic experiments (Lu et al., 2020). In order to understand the relationship between the Rln3-expressing and NMB-expressing neuron populations in this area, we used dual-label FISH for Rln3 and NMB mRNA in serial sections throughout their entire extent of expression in the pons (Fig. 3). In the rostral and central parts of the NI, Rln3 and NMB transcripts were co-localized in most NI neurons (Fig. 3*A–C*). A subset of cells expressing NMB alone were observed, but cells expressing only Rln3 were rare at any level. At the caudal pole of the NI, at a level where the Rln3 neurons become sparse, neurons detected in the CG expressed only NMB (Fig. 3*D*). These NMB-only neurons continued to the caudal end of the pons, to the level of the PDTg, in areas outside the NI in standard atlases (Fig. 3*E*,*F*). We conclude that Rln3-expressing neurons are a subset of the NMB-expressing neurons. Within the CG, the extent of Rln3 expression corresponds best to the anatomic region usually designated as the NI, while NMB is expressed more extensively in the pontine tegmentum.

**Figure 4.**
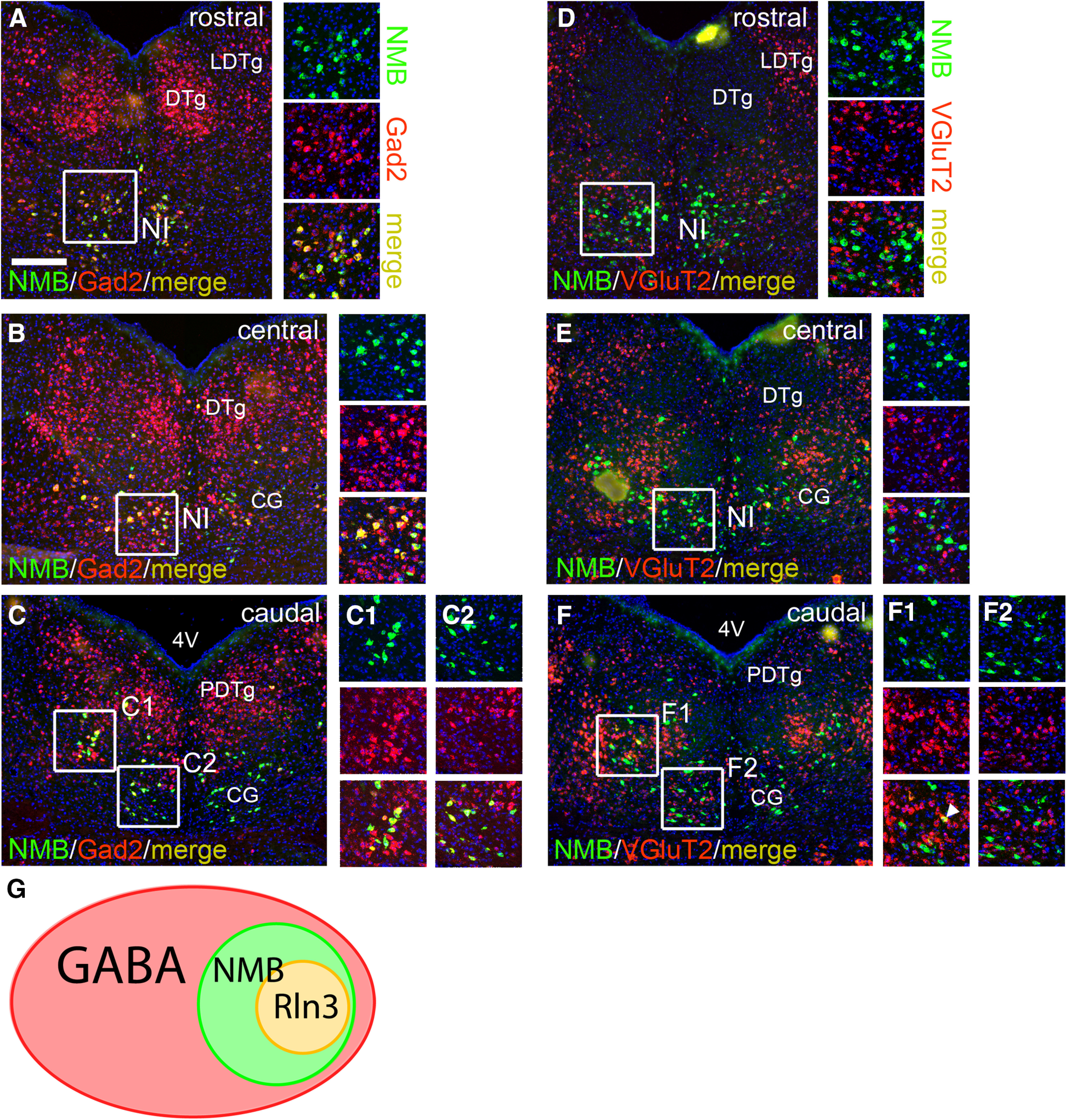
Fast neurotransmitter markers in NMB-expressing neurons in the pontine tegmentum. FISH was used to examine co-expression of NMB mRNA and GAD2 or VGluT2 in the rostral, central, and caudal NI, corresponding to bregma −5.34, bregma −5.40/−5.52 and bregma −5.68 in a standard atlas. Z-stacks of confocal images of the cells in the boxed area of each panel are shown at right. ***A–C***, NMB and GAD2 expression. No NMB-expressing neurons were identified which did not also express GAD2. ***C1***, ***C2***: top panel, NMB signal; middle panel, Gad2 signal; bottom panel, merged signal. ***D–F***, NMB and VGluT2 expression. ***F1***, ***F2***: top panel, NMB signal; middle panel, VGluT2 signal; bottom panel, merged signal. No cells with clear co-expression of NMB and VGluT2 were identified. A rare case of convergent signal in the confocal image appears to result from overlapping cellular profiles in a densely packed area, not cellular co-expression (arrow, ***F1***). ***G***, Schematic of the relationship of the neurotransmitter phenotypes in the pontine tegmentum, derived from combined data in Figures 3, 4. 4V, fourth ventricle; CG, central pontine gray; DTg, dorsal tegmental nucleus; LDTg, laterodorsal tegmental nucleus; NI, nucleus incertus; PDTg, posterodorsal tegmental nucleus. Scale bar: 200 μm (***A***).

All or nearly all NI-Rln3 neurons express GABAergic markers and are presumed to be GABAergic (Fig. 1*K*; Ma et al., 2007). However, intermingled glutamatergic neurons have also been identified in the NI (Cervera-Ferri et al., 2012). In order to ascertain the fast neurotransmitter in NMB-expressing neurons in the pontine tegmentum, we used dual-label FISH for NMB mRNA together with either GAD2 or VGluT2, the principal subcortical glutamate transporter, throughout the extent of the NI (Fig. 4). GAD2 mRNA expression was extensive in the pontine tegmentum, including the DTg, CG and NI (Fig. 4*A–C*). Confocal imaging for cellular co-localization of the markers demonstrated that all identified NMB-expressing neurons also expressed GAD2. VGluT2-expressing neurons were present in areas adjacent to the NI, but only rare colocalization with NMB was observed, probably representing cellular overlap in densely packed areas (Fig. 4*D–F*). We conclude that all, or nearly all, of the NMB neurons in the pons are GABAergic. Thus, the NMB-expressing neurons are a subset of a very large set of GABAergic neurons in the pons, and Rln3-expressing neurons are in turn a subset of the NMB-expressing cells, largely restricted to the NI and the adjacent pontine raphe nucleus (PnR; Fig. 4*G*).

### Anterograde tracing of mesencephalic Rln3-expressing neurons

**Figure 5.**
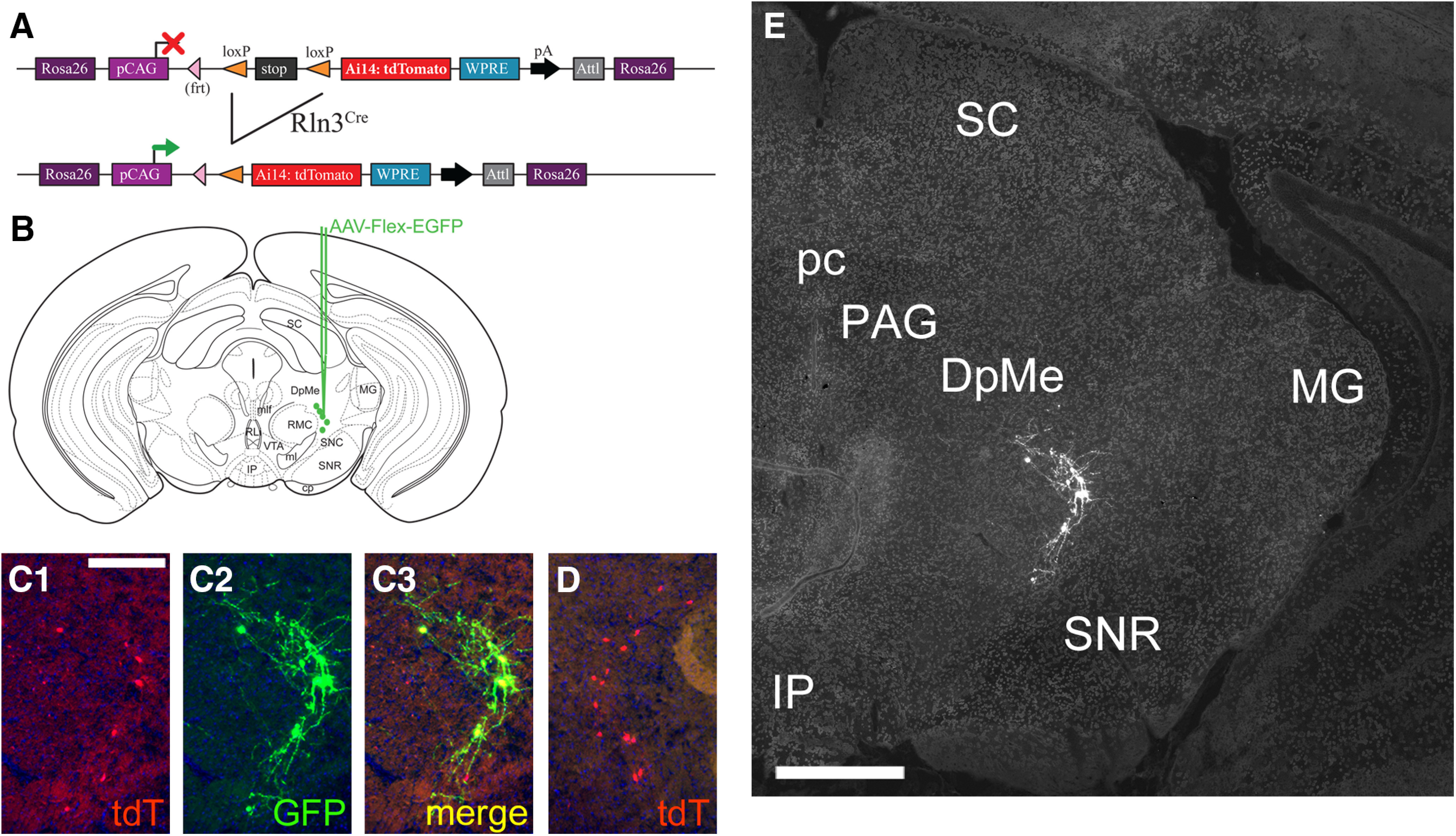
Anterograde viral labeling of Rln3 neurons in the deep mesencephalic area. ***A***, Strategy for genetic labeling of *Rln3^Cre^* mice bearing the *Gt(ROSA)26*-targeted tdTomato reporter allele Ai14. Genetic labeling of Rln3^tdT^ neurons in this way allows assessment of the efficiency of viral labeling. ***B***, Injection of AAV: FLEX-GFP into the deep mesencephalic area, dorsal to the substantia nigra, at a level corresponding to bregma −3.4 in a standard atlas (Paxinos and Franklin, 2001). Targeted coordinates: AP: −3.50, ML: 1.15, DV: 3.00. ***C***, Expression of genetically expressed tdTomato, virally expressed EGFP, and both markers together in the injected area. ***C1***, tdTomato signal; ***C2***, GFP signal; ***C3*** merged images. ***D***, *Rln3^Cre^*-induced tdTomato expression on the side contralateral to the injection. ***E***, Labeled cell bodies and fibers in the deep mesencephalic area. Labeled fibers from *Rln3^Cre^* neurons in the DpMe were not detected outside the mesencephalon. DpMe, deep mesencephalic area; IP, interpeduncular nucleus; MG, medial geniculate; PAG, periaqueductal gray; pc, posterior commissure; SC, superior colliculus; SNR, substantia nigra, pars reticulata. Scale bars: 200 μm (***C***) and 500 μm (***E***).

The neuroanatomy of the ascending Rln3 system has been described in the mouse using immunostaining for Rln3-containing fibers (Smith et al., 2010), but this method does not determine the source of these fibers, since multiple brain nuclei express Rln3. In order to better understand the relationship of Rln3-expressing neurons to their targets, we injected Cre-dependent tract-tracing AAVs expressing fluorescent markers into each brain region containing Rln3-expressing neurons, including the DpMe, the PAG, and the NI. In order to trace the efferents of *Rln3^Cre^* neurons in the DpMe, we injected transgenic mice genetically engineered to express a tdTomato reporter in all *Rln3^Cre^* neurons (Fig. 5*A*), with a Cre-dependent AAV encoding axonally targeted EGFP (AAV-Flex-EGFP; Fig. 5*B*), resulting in very efficient unilateral labeling of this cell group (Fig. 5*C*,*D*). Labeling of the DpMe cell group revealed very limited projections within the mesencephalon, medial and lateral to the injection site (Fig. 5*E*). Labeled fibers were not detected in other areas known to receive Rln3 inputs, such as the cerebral cortex, septum, lateral hypothalamus (LH), and raphe (data not shown).

**Figure 6.**
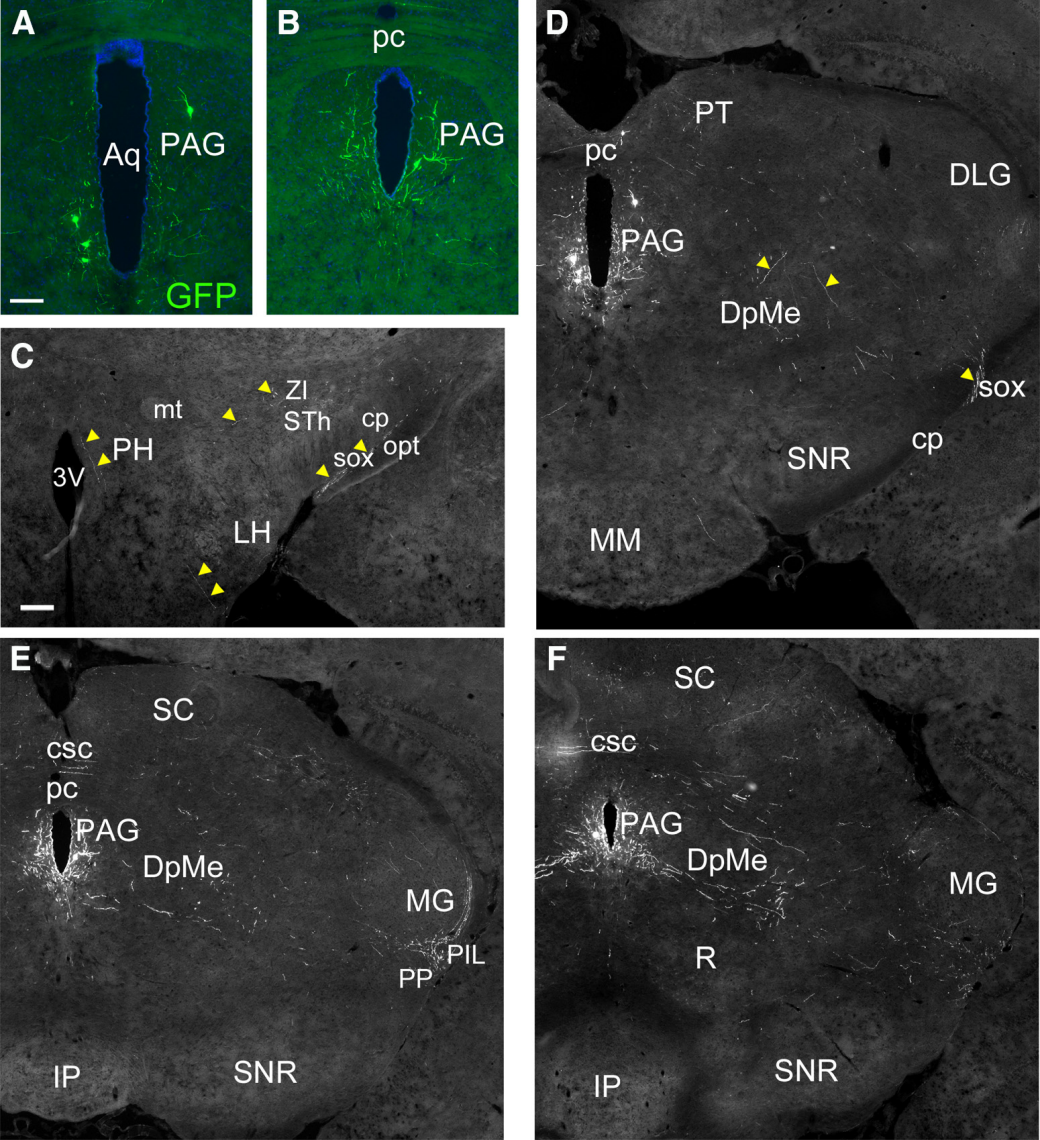
Anterograde viral labeling of Rln3 neurons in the mesencephalic PAG. ***A***, ***B***, Cell bodies of *Rln3^Cre^* neurons in the PAG labeled by injection of AAV:FLEX-GFP. Targeted coordinates: AP: −2.80, ML: 0.1, DV: 3.00. ***C***, Sparse labeled fibers in the hypothalamic area and supraoptic decussation (arrows), at a level corresponding to bregma −2.0 in a standard atlas. ***D***, Labeled fibers in the mesencephalon and supraoptic decussation (arrows; same section as in ***A***), bregma −2.8. ***E***, Labeled fibers in the mesencephalon and inferior to the MG (same section as in ***B***), bregma −3.1. ***F***, Labeled fibers in the mesencephalon and inferior to the MG, bregma −3.4. 3V, third ventricle; Aq, aqueduct; cp, cerebral peduncle; csc, commissure of the superior colliculus; DLG,; DpMe, deep mesencephalic area; IP, interpeduncular nucleus; LH, lateral hypothalamus; MG, medial geniculate; MM, medial mammillary nucleus; mt, mammillothalamic tract; opt, optic tract; PAG, periaqueductal gray; pc, posterior commissure; PH, posterior hypothalamus; PIL, posterior intralaminar thalamic nucleus; PP, peripeduncular nucleus; PT, pretectum; R, red nucleus; SC, superior colliculus; sox, supraoptic decussation; SNR, substantia nigra, pars reticulata; STh, subthalamic nucleus; ZI, zona incerta. Scale bars: 100 μm (***A***) and 200 μm (***C***).

Injection of AAV-Flex-EGFP was also used to label *Rln3^Cre^* neurons in the PAG (Fig. 6*A*,*B*). Generally, five or fewer labeled cells were observed per section in this area, but this appears to reflect the actual sparse nature of this cell group (Fig. 2*D*). Injection of PAG resulted in a few labeled fibers in the LH, posterior hypothalamus (PH), and zona incerta (ZI), but labeled fibers were not observed in the septum, hippocampus, or neocortical areas (Fig. 6*C*; data not shown). Predominantly, labeled fibers were observed within the deep mesencephalic area, frequently running mediolaterally within the coronal plane of section (Fig. 6*D–F*). The densest innervation was observed in the posterior intralaminar thalamic nucleus (PIL), an area just inferior to the medial geniculate (MG; Fig. 6*E*,*F*); these fibers appeared to condense to join the supraoptic decussation (sox; Fig. 6*C*,*D*), which is known to connect the MG hemispheres. No descending projections were identified for these neurons. These findings show that the Rln3-expressing PAG neurons are part of a very specific mesencephalic pathway, of unknown function.

### Anterograde tracing of NI-Rln3-expressing neurons

**Figure 7.**
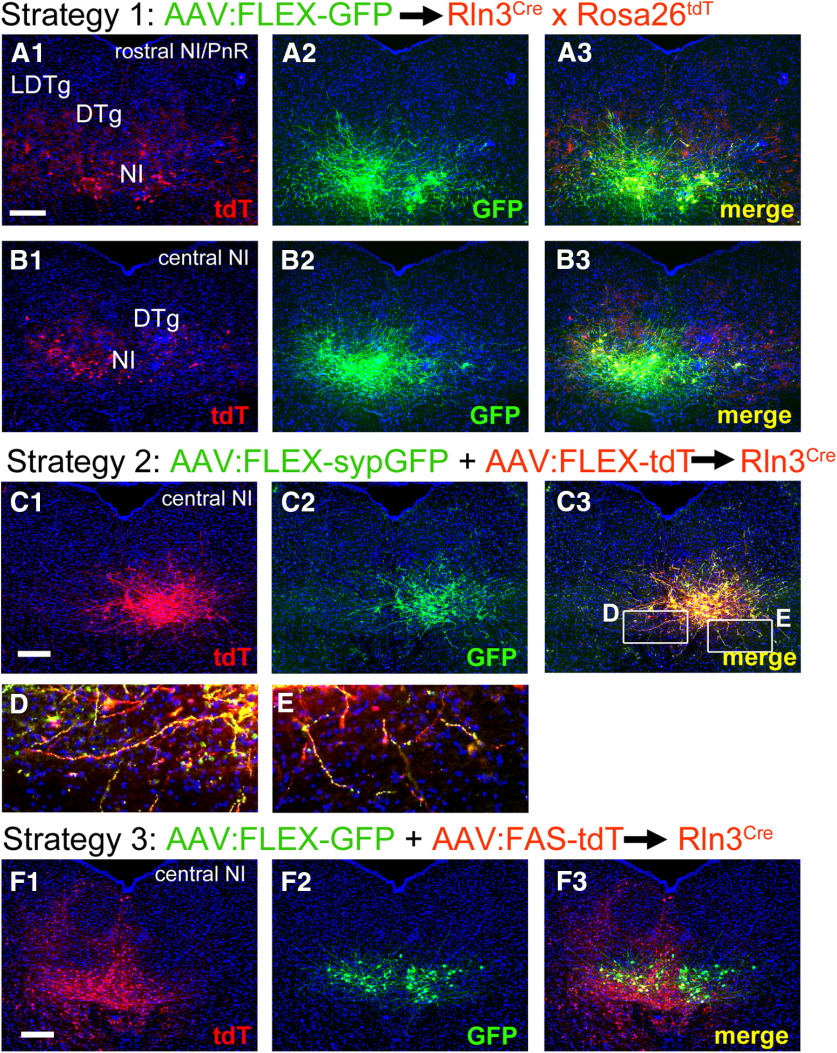
Anterograde viral labeling strategies for the projections of NI/Rln3 neurons. ***A***, ***B***, Injection of AAV:FLEX-GFP into *Rln3^Cre^* mice bearing the *Gt(ROSA)26*-targeted tdTomato reporter allele Ai14. ***A1***,***B1***, tdTomato signal; ***A2***,***B2***, GFP signal; ***A3***,***B3***, merged image. This genetic labeling of Rln3^tdT^ neurons allows assessment of the efficiency of viral labeling. The injected AAV efficiently labels the entire rostrocaudal extent of the NI, with labeling predominantly on the left. Targeted coordinates: AP: −5.40, ML: 0.10, DV: 3.95. Anterograde tracing data appear in Figures 8, 9. ***C–E***, Co-injection of AAV:FLEX-sypGFP and AAV:FLEX -tdTomato into *Rln3^Cre^* mice. Expression is largely restricted to the right side. ***C1***, tdTomato signal; ***C2***, GFP signal; ***C3***, merged image. In ***D***, ***E***, individual labeled fibers can be seen in red, with presumptive synaptic areas labeled in green. Targeted coordinates: AP: −5.40, ML: 0.10, DV: 4.15. Anterograde tracing data appear in Figures 10, 11. ***F***, Injection of AAV:FLEX-GFP and AAV:FAS-tdTomato into *Rln3^Cre^* mice. ***F1***, tdTomato signal; ***F2***, GFP signal; ***F3***, merged image. In this “Cre-on, Cre-off” strategy, the expression cassette in the FAS-tdTomato virus is inactivated rather than activated by Cre recombinase. This allows the specific labeling of the *Rln3^Cre^*-negative neurons in the injected area, and allows the efferents of the Cre-expressing and Cre-non-expressing NI neurons to be distinguished. Labeling of *Rln3^Cre^* neurons is bilateral, but labeling of the surrounding area with FAS-tdT is more extensive on the left. In some Cre-expressing neurons, the inactivation of FAS-tdT was incomplete, and these neurons appear yellow in the merged version. Targeted coordinates: AP: −5.40, ML: 0.10, DV: 4.15. Anterograde tracing data appear in Figure 12. DTg, dorsal tegmental nucleus; LDTg, laterodorsal tegmental nucleus; NI, nucleus incertus. Scale bar: 200 μm.

Three AAV-mediated strategies were used to map the projections of *Rln3^Cre^* neurons in the NI. In strategy 1, we injected transgenic mice genetically engineered to express a tdTomato reporter in all *Rln3^Cre^* neurons with AAV-FLEX-EGFP, as described for mapping the DpMe and PAG (Fig. 7*A*,*B*). In strategy 2, we injected a mixture of two Cre-activated AAVs, one expressing tdTomato that predominantly labeled cell bodies, axons, and fibers of passage, and the other expressing a sypGFP fusion protein that predominantly labeled presynaptic terminals (FLEX-tdT and FLEX-sypGFP; Fig. 7*C*). Thus, where fibers labeled with both markers run in the plane of section through an area of synaptic contact, they resemble “green beads on a red string” (Fig. 7*D*,*E*). In strategy 3, we used a “Cre-on, Cre-off” strategy to trace NI efferents, combining a FLEX-GFP virus activated by Cre, and a FAS-tdTomato virus inactivated by Cre, injected into *Rln3^Cre^* mice (Fig. 7*F*; Saunders et al., 2012). In such a strategy, projections from *Rln3^Cre^* neurons should be labeled with GFP, and those from Rln3-negative NI neurons labeled with tdT, allowing a direct comparison of the areas receiving efferents from Rln3-expressing and non-expressing neurons in the CG.

**Figure 8.**
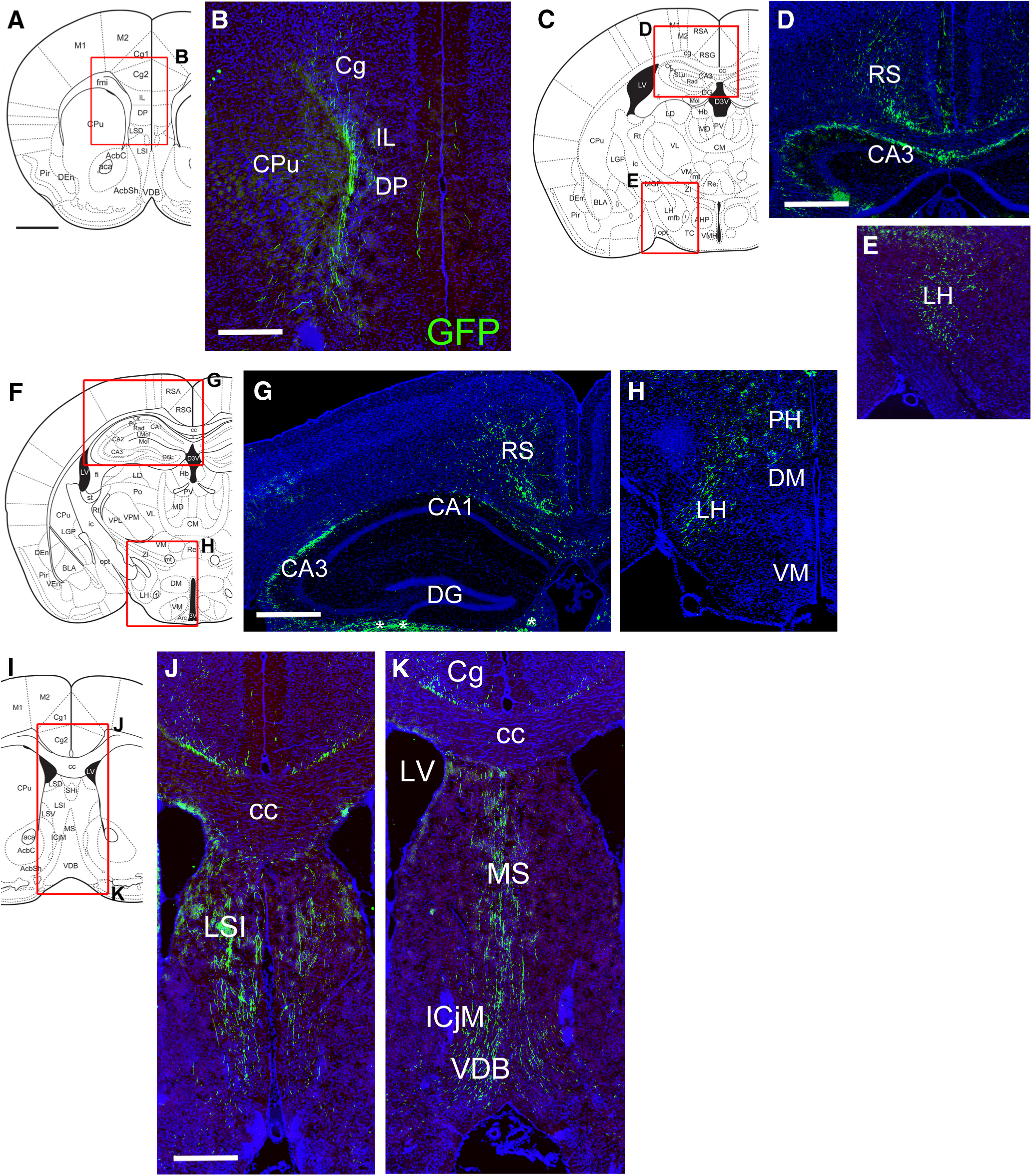
Projections of NI/Rln3 neurons to the rostral CNS using AAV: FLEX-GFP. The injected area appears in Figure 7*A*,*B*. ***A*, *B***, Labeled fibers appear in the prelimbic and cingulate cortex, at a level corresponding to bregma 1.34 in a standard atlas (Paxinos and Franklin, 2001). ***C***, ***E***, Fibers in the RS and hippocampus (***D***) and the rostral LH (***E***), bregma −1.06. ***F*, *H***, Labeled fibers in the RS and hippocampus (***F***) and caudal LH (***H***), bregma −1.70. ***I***, ***K***, Fibers in the septum and diagonal band, bregma 0.98. Because of the tilt of the sections, (***J***) corresponds to the dorsal level of the schematic, and (***K***) corresponds to the ventral level of the schematic. CA1, CA3, hippocampal regions CA1, CA3; cc, corpus callosum; Cg, cingulate cortex; CPu, caudate/putamen; DG, dentate gyrus; DM, dorsomedial hypothalamus; DP, dorsal peduncular cortex; ICjM, islands of Calleja; IL, infralimbic cortex; LH, lateral hypothalamus; LSI, lateral septal nucleus, intermediate part; LV, lateral ventricle; MS, medial septum; PH, posterior hypothalamus; RS, retrosplenial cortex; VDB, ventral diagonal band; VM, ventromedial hypothalamus. Scale bars: 1 mm (***A***, ***C***, ***F***, ***I***) and 500 μm (***B***, ***D***, ***G***, ***J***).

**Figure 9.**
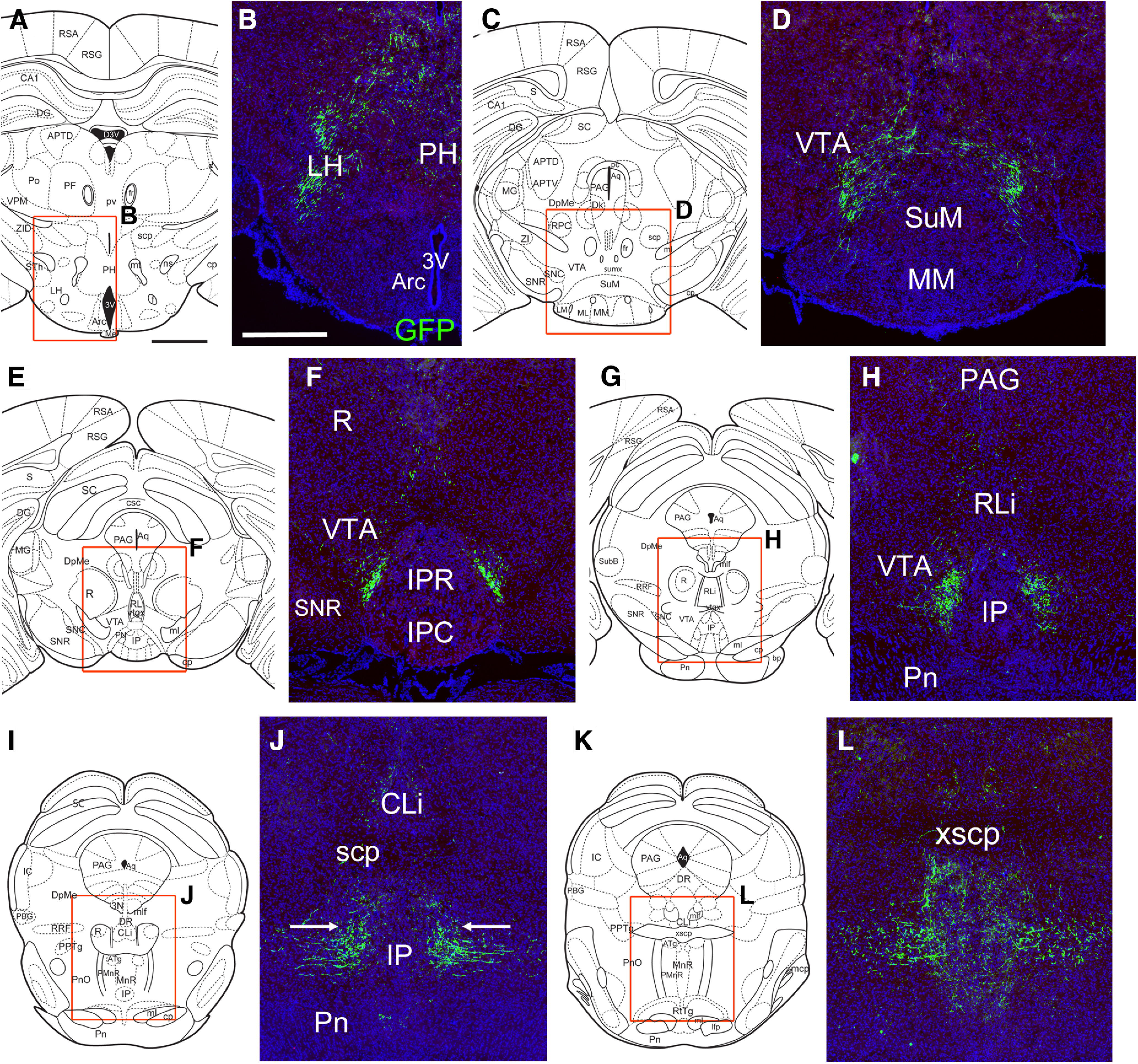
Projections of NI/Rln3 neurons to the mesopontine tegmentum and raphe using AAV:FLEX-GFP. ***A***, ***B***, Labeled fibers in the LH and PH, at a level corresponding to bregma −2.30 in a standard atlas. ***C***, ***D***, Fibers in and near the VTA, bregma −2.92. ***E***, ***F***, Fibers in the tegmentum, adjacent to the rostral interpeduncular nucleus, bregma −3.28. ***G***, ***H***, Projections in the tegmentum, adjacent to the caudal interpeduncular nucleus, bregma −3.40. ***I***, ***J***, Fibers in the tegmentum, adjacent to the rostral median raphe, bregma −3.88. At this level, fibers of the ascending NI tract in the pontine reticular nucleus (PnO) move toward the midline (arrows). ***K***, ***L***, Fibers in the median raphe and PnO, bregma −4.48. 3V, third ventricle; Arc, arcuate nucleus; CLi, caudal linear nucleus of raphe; IP, interpeduncular nucleus: IPC, caudal part; IPR, rostral part; LH, lateral hypothalamus; MM, medial mammillary nucleus; PAG, periaqueductal gray; PH, posterior hypothalamus; Pn, pontine nucleus; PnO, pontine reticular nucleus; R, red nucleus; RLi, rostral linear nucleus of raphe; scp, superior cerebellar peduncle; SnR, substantia nigra, pars reticulata; SuM, supramammillary nucleus; VTA, ventral tegmental area; xscp, decussation of the superior cerebellar peduncle. Scale bar: 1 mm (***A***) and 500 μm (***B***).

Injection of AAV-FLEX-GFP into Rln3^Cre^ mice was used to map the ascending NI-Rln3 system throughout the CNS. In the neocortex, fibers were prominent in the infralimbic (IL) and dorsal peduncular (DP) areas (Fig. 8*A*,*B*), and in the RS (Fig. 8*C*,*D*,*F*,*G*). Functional studies of the NI have focused on its input to the septohippocampal system (Martínez-Bellver et al., 2015, 2017; Szőnyi et al., 2019; Lu et al., 2020). In the hippocampus, labeled fibers were prominent in CA3, and sparse in CA1, and lacking in the DG (Fig. 8*C*,*D*,*F*,*G*). In the septal complex (Fig. 8*I–K*), labeled fibers were prominent in the intermediate part of the lateral septum (LSI), the MS and the ventral limb of the diagonal band (VDB). In the diencephalon, fibers were noted in the LH and PH, but not in ventral hypothalamic nuclei (Figs. 8*E*,*F*,*H*, 9*A*,*B*). At the transition from the hypothalamus to the ventral tegmental area (VTA), strongly labeled fibers were observed between the VTA and supramammillary nucleus (SuM; Fig. 9*C*,*D*). At more caudal levels, these fibers run between the VTA and interpeduncular nucleus (IP; Fig. 9*E–J*). Since all of the ascending fibers from NI-Rln3 neurons must pass through this area, we hypothesized that many of these are fibers of passage, and examined this using the synaptic labeling strategy, as described below. At the level of the decussation of the superior cerebellar peduncle (xscp), fibers were seen throughout the median raphe (MnR) and paramedian raphe (PMnR; Fig. 9*K*,*L*).

**Figure 10.**
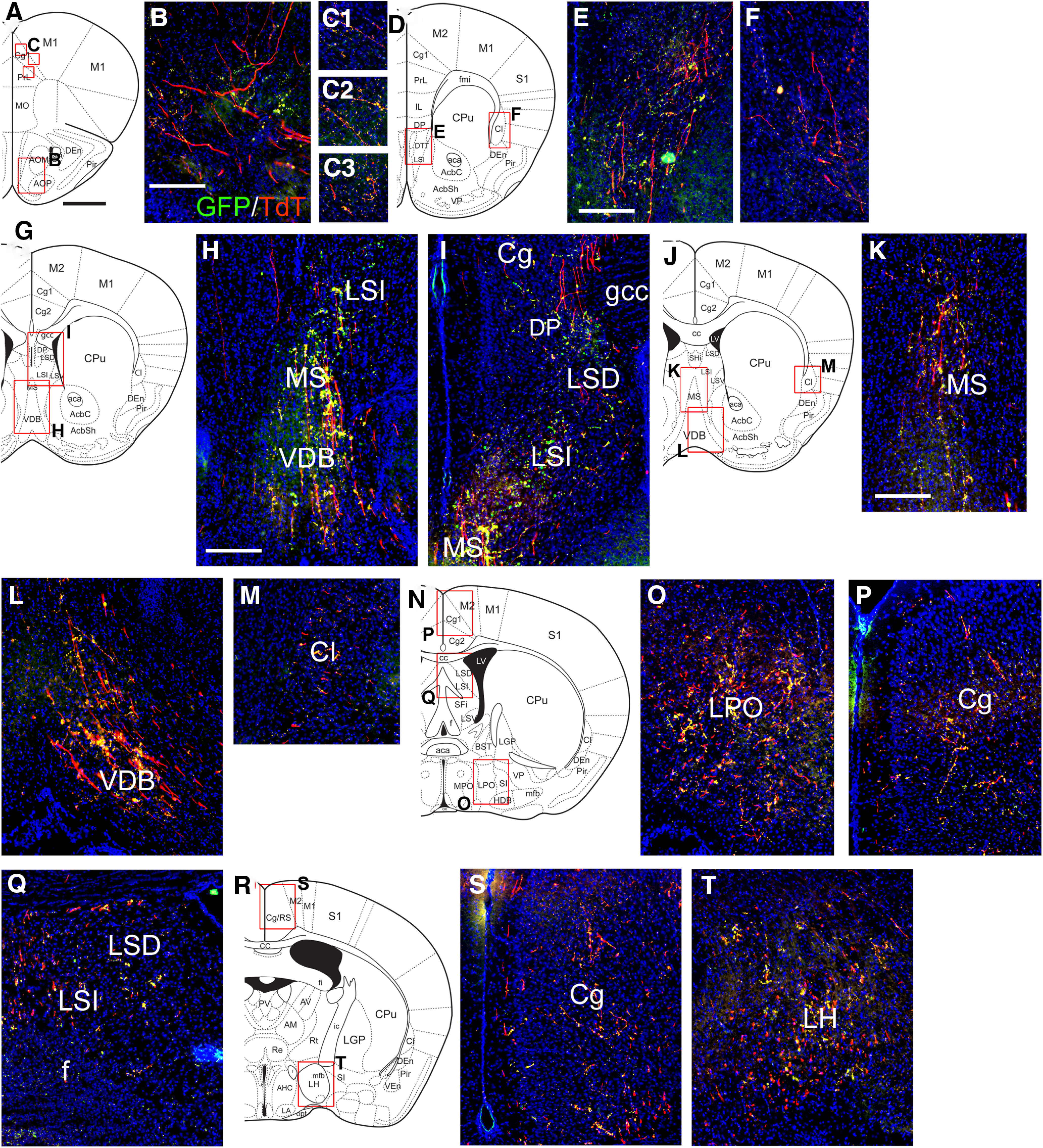
Projections of NI/Rln3 neurons to the rostral CNS using co-injection of AAV:FLEX-sypGFP and AAV:FLEX-tdTomato. The injected area appears in Figure 7*C–E*. ***A–C***, Fibers and sparse presumptive synapses in the olfactory area (***B***), and in the cingulate cortex (C1-3), at a level corresponding to bregma 2.2 in a standard atlas. ***D–F***, Fibers and sparse synapses in the rostral septum (***E***) and claustrum (***F***), bregma 1.54. ***G–I***, Fibers and synapses in the medial and lateral septum, bregma 1.18. ***J–M***, Fibers and synapses in the MS (***K***), diagonal band (***L***), and claustrum (***M***), bregma 0.98. ***N–Q***, Fibers and synapses in the lateral preoptic nucleus (***O***), cingulate cortex (***P***), and caudal septum (***Q***), bregma 0.02. **(*R–T*)** Fibers and synapses in the cingulate cortex (***S***) and LH (***T***), bregma −0.70. Cg, cingulate cortex; Cl, claustrum; CPu, caudate/putamen; DG, dentate gyrus; DM, dorsomedial hypothalamus; DP, dorsal peduncular cortex; f, fornix; gcc, genu of the corpus callosum; LH, lateral hypothalamus; LPO, lateral preoptic area; LS, lateral septum, LSD, dorsal part, LSI intermediate part; MS, medial septum; VDB, ventral diagonal band; VM, ventromedial hypothalamus. Scale bars: 1 mm (all atlas views) and 200 μm (all fluorescence views).

**Figure 11.**
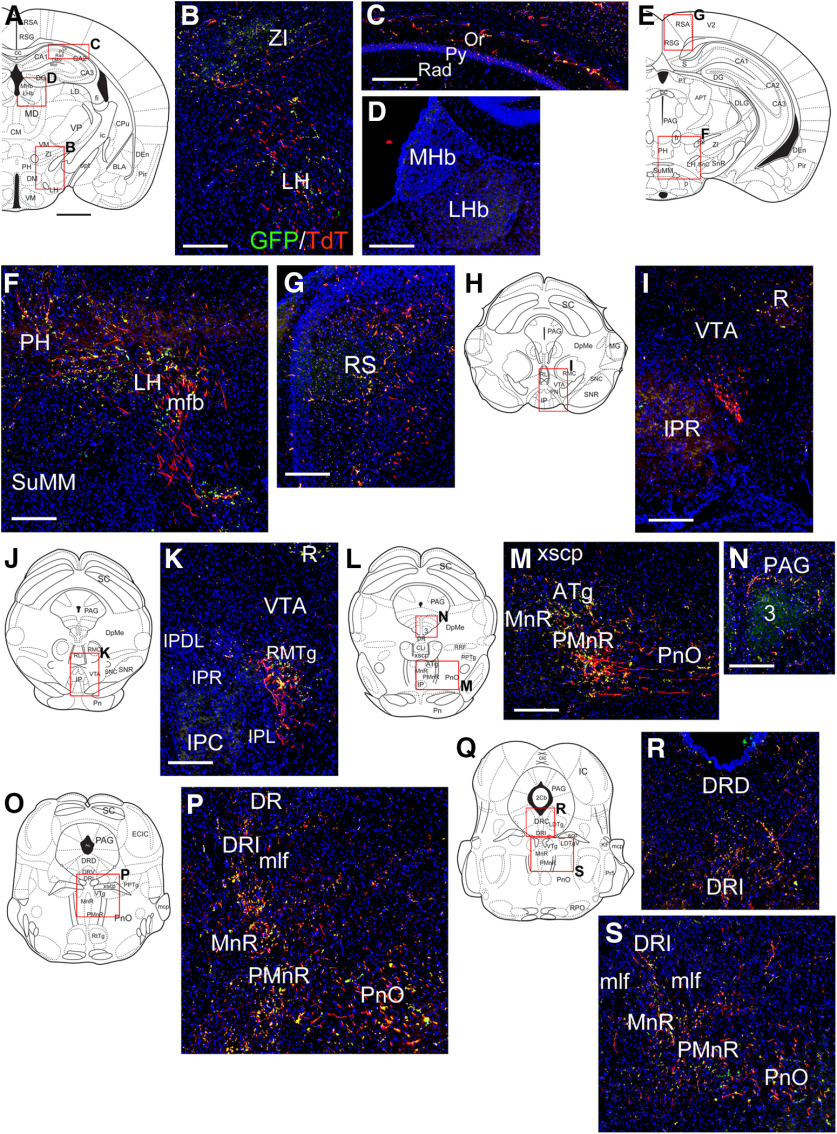
Projections of NI/Rln3 neurons to the mesopontine tegmentum and raphe using co-injection of AAV:FLEX-sypGFP and AAV:FLEX-tdTomato. ***A–D***, Fibers and presumptive synapses in the LH (***B***) and hippocampus (***C***), at a level corresponding to bregma −1.70 in a standard atlas. Projections were not detectable in the medial or lateral habenula (***D***). ***E–G***, Fibers and projections in the caudal part of the LH and the PH (***F***) and the RS (***G***), bregma −2.70. ***H***, ***I***, Fibers of passage lying between the interpeduncular nucleus and ventral tegmental nucleus, bregma −3.40. The label is very sparse within the IP and VTA. Green signal in the red nucleus is an autofluorescence artifact. ***J***, ***K***, Fibers and synapses in the rostromedial tegmental nucleus, adjacent to the caudal part of the interpeduncular nucleus, bregma −3.88. Signal in the red nucleus in ***K*** is autofluorescence. ***L–N***, Fibers and synapses in the median raphe/paramedian raphe (***M***) and PAG (***N***), bregma −4.16. ***O***, ***P***, Fibers and synapses in the MnR/PMnR, and the pontine reticular nucleus, bregma −4.72. At this level, ascending projections of NI/Rln3 neurons run in the plane of section from a lateral tract in PnO to a more medial tract, to eventually reach the position adjacent to IP shown in ***I***, ***K***. Sparse synapses are also observed in the dorsal raphe. ***Q–S***, Fibers and synapses in the DR (***R***), MnR/PMnR, and PnO (***S***), bregma −4.96. Sections caudal to this level are included in the images of the injection site. 3, oculomotor nucleus; ATg, anterior tegmental nucleus, also identified as the caudal RMTg (Quina et al., 2015); CA1, CA1 of hippocampus: oriens layer, Or, pyramidal layer, Py, stratum radiatum, Rad; DR, dorsal raphe, DRD, dorsal part, DRI, interfascicular part; IP, interpeduncular nucleus: IPC, caudal part; IPDL, dorsolateral part, IPL, lateral part, IPR, rostral part; LH, lateral hypothalamus; LHb, lateral habenula; mfb, medial forebrain bundle; MHb, medial habenula; mlf, medial longitudinal fasciculus; PAG, periaqueductal gray; PH, posterior hypothalamus; MnR, median nucleus raphe; PMnR, paramedian nucleus raphe; PnO, pontine reticular nucleus; R, red nucleus; RMTg, Rostromedial tegmental nucleus; RS, retrosplenial cortex; SuMM, supramammillary nucleus, median part; VTA, ventral tegmental area; xscp, decussation of the superior cerebellar peduncle; ZI, zona incerta. Scale bars: 1 mm (all atlas views) and 200 μm (all fluorescence views).

In order to better distinguish areas in which NI-Rln3 neurons potentially make synaptic connections from those containing fibers of passage, we examined the brain areas receiving inputs from NI-Rln3 neurons using AAV-FLEX-mediated expression of GFP fused to the synaptic protein synaptophysin (sypGFP) plus the cytoplasmic/axonal label tdTomato encoded by a separate AAV, both activated by *Rln3^Cre^* (strategy 2). In the neocortex, tdT labeled fibers and punctate sypGFP labeling were observed in the prelimbic cortex (PrL; Fig. 10*A*,*C*), the cingulate cortex (Fig. 10*A*,*C*,*N*,*P*,*R*,*S*), the DP, and the IL (Fig. 10*D*,*E*; data not shown). Labeled fibers and sparse synapses were also seen in the olfactory bulb (Fig. 10*A*,*B*). In prior studies using immunohistochemical methods, Rln3 fibers have been identified in the claustrum (Smith et al., 2010); although tdT labeled fibers were evident there, sypGFP expression was low (Fig. 10*D*,*F*,*J*,*M*). Strong sypGFP labeling was observed in every part of the septal complex including the LSI, the dorsal lateral septum (LSD), the MS, and the VDB (Fig. 10*G–L*,*N*,*Q*). Fibers in CA3 of the hippocampus were also accompanied by punctate sypGFP expression, implying synaptic contacts (Fig. 11*A*,*C*). Overall, the fiber and synaptic labeling in the septal nuclei was markedly denser than that observed in hippocampus. In the hypothalamus, fibers were accompanied by sypGFP labeling in the lateral preoptic nucleus (LPO; Fig. 10*N*,*O*), which is continuous with the LH at more caudal levels (Figs. 10*R*,*T*, 11*A*,*B*,*E*,*F*). Fiber and synaptic labeling was noted in the RS, continuous with cortical afferents seen at more rostral levels (Fig. 10*G*). Fiber terminals were sparse in the VTA and IP, but tdT-labeled fibers of passage appeared just lateral to the IP (Fig. 11*H–K*), marking the only identifiable ascending tract containing the NI efferents. Synaptic labeling was also observed throughout the MnR/PMnR (Fig. 11*M–S*), and in the inferior part of the dorsal raphe (Fig. 11*Q*,*R*). A summary of the areas in which the NI-Rln3 neurons make potential synaptic contacts is presented in Table 1.

**Table 1:** Summary of areas receiving potential synaptic input from the NI

Area	Abbreviation	sypGFP	Reference
Olfactory area			
Anterior olfactory n.	AO	+	Figure 10*B*
Telencephalon			
Cerebral cortex			
Cingulate cortex	Cg	++	Figure 10*C,P,S*
Claustrum	Cl	+	Figure 10*F,M*
Dorsal peduncular cortex	DP	+	Figure 10*I*
Infralimbic cortex	IL	+	
Orbital cortex, medial	MO	+	
Prelimbic cortex	Prl	+	Figure 10*C*
Retrosplenial cortex	RS	++	Figure 11*G*
Hippocampus			
CA1 field, oriens layer	CA1	+	Figure 11*C*
CA2 field, oriens layer	CA2	+	Figure 11*C*
CA3 field, oriens layer	CA3	+	
Dentate gyrus	DG	+	
Striatum/pallidum			
Dorsal tenia tecta	DTT	++	Figure 10*E*
Nucleus diagonal band			
Horizontal limb	HDB	++	
Vertical limb	VDB	+++	Figure 10*H,L*
Septum			
Lateral septal n., dorsal	LSD	+	Figure 10* I,Q*
Lateral septal n., intermediate	LSI	++	Figure 10*H,L,Q*
Medial septal n.	MS	+++	Figure 10*H,I,K*
Septofimbrial n.	SFi	+	
Triangular septal n.	TS	+	
Diencephalon			
Thalamus			
Zona incerta	ZI	+	Figure 11*B*
Hypothalamus			
Lateral hypothalamic area	LH	+++	Figures 10*T*, 11*B,F*
Posterior hypothalamic area	PH	++	Figure 11*F*
Preoptic area, lateral	LPO	+++	Figure 10*O*
Supramammillary n.	SuM	+	
Mesencephalon			
Dorsal raphe n., dorsal part	DRD	+	Figure 11*R*
Dorsal raphe n., inferior part	DRI	++	Figure 11*P,R,S*
Interpeduncular nucleus^1^	IP	+/−	Figure 11*I,K*
Rostromedial tegmental n.^2^	RMTg	+++	Figure 11*K*
Ventral tegmental area^3^	VTA	+/−	Figure 11*I,K*
Periaqueductal gray	PAG	+	Figure 11*N*
Rhombencephalon			
Central gray, pontine	PnO	++	Figure 11*M,P,S*
Median raphe n.	MnR	+	Figure 11*M,P,S*
Nucleus incertus (cell bodies)	NI/O	+++	Figure 7
Paramedian raphe n.	PMnR	+++	Figure 11*M,P,S*
Anterior tegmental n.^4^ (caudal RMTg)	ATg	++	Figure 11*M*

**Figure 12.**
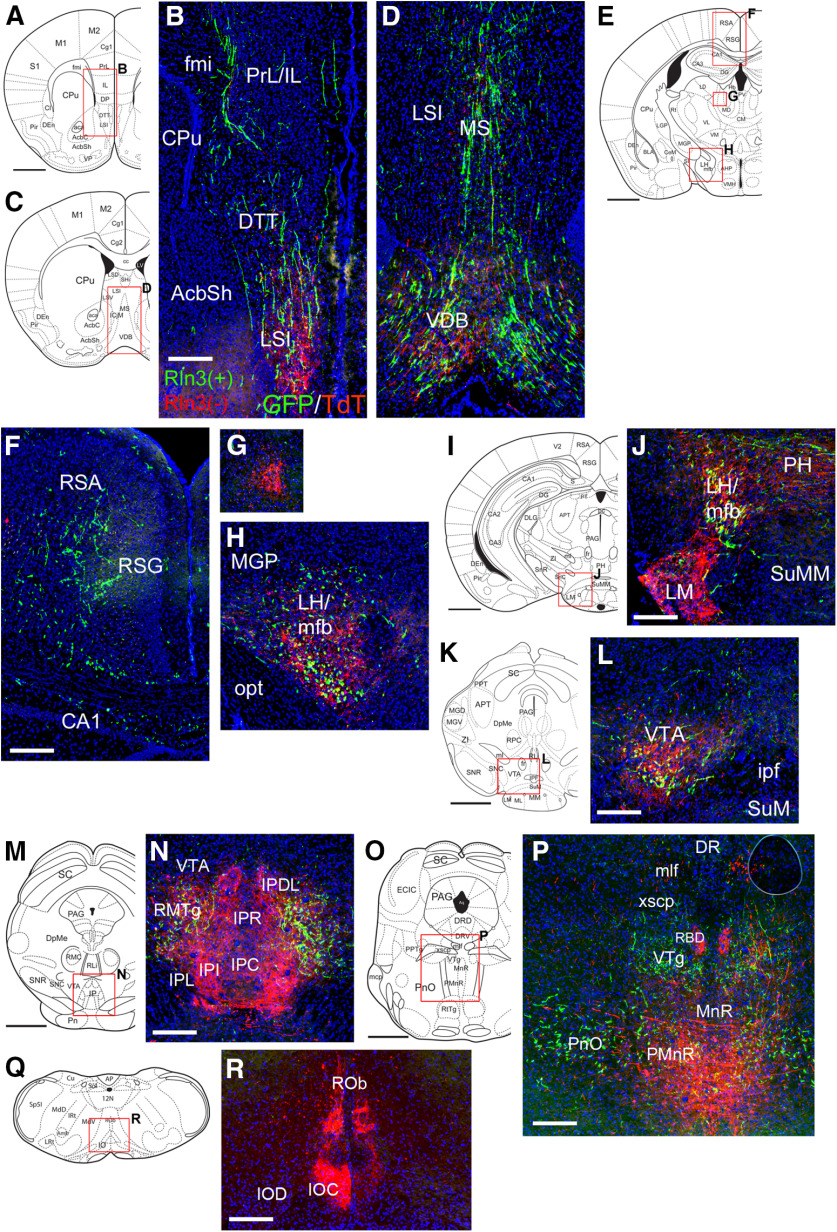
Projections of NI Rln3-positive and Rln3-negative neurons using co-injection of AAV:FLEX-GFP and AAV: FAS-tdTomato. The injected area appears in Figure 7*F*. ***A***, ***B***, Projections to the limbic cortex and rostral septum, at a level corresponding to bregma 1.54 in a standard atlas. ***C***, ***D***, Projections to the MS and ventral diagonal band, bregma 0.98. In ***B***, ***D***, it appears that fibers of Rln3(+) and Rln3(−) neurons project to the ventral parts of the septum, but only Rln3(+) neurons project to the dorsal septum and limbic cortex. ***E–H***, Projections to the RS and hypothalamus, bregma −1.22. Only Rln3(+) neurons appear to project to the RS (***F***); Rln3(−) neurons appear to innervate a unique territory in the medial thalamus (***G***); Rln3(+) and Rln3(−) neurons both innervate the LH/medial forebrain bundle (***H***). ***I***, ***J***, Projections to the caudal hypothalamus, bregma −2.70. Fibers from Rln3(−) neurons predominate in the LM, while Rln3(+) and Rln3(−) neurons both innervate the LH and PH. ***K***, ***L***, Projections to the area at the transition from the caudal LH to the rostral VTA, bregma −3.08. Fibers from Rln3(+) and Rln3(−) neurons have a similar distribution. Fibers of passage may predominate here since synaptic labeling of the VTA from Rln3(+) neurons is sparse (Fig. 11*I*). ***M***, ***N***, Projections to the caudal midbrain tegmentum, bregma −3.88. Rln3(+) and Rln3(−) neurons both innervate the rostromedial tegmental nucleus, while only projections of Rln3(−) neurons terminate within the interpeduncular nucleus. NI fibers are sparse in the VTA at this level. ***O***, ***P***, Projections to the raphe, bregma −4.72. Fibers from Rln3(+) neurons predominate in the pontine reticular nucleus; fibers of both Rln3(+) and Rln3(−) neurons are found in the MnR/PMnR, but in a somewhat different distribution in this diverse area. The rhabdoid nucleus is innervated exclusively by Rln3(−) neurons. Fibers from Rln3(−) neurons can also be seen ascending more dorsally in the mlf. ***Q***, ***R***, Projections to the IO, bregma −7.46. Labeled fibers are seen only in the dorsal, central part of the IO, and originate exclusively in Rln3(−) neurons. AcbSh, accumbens nucleus, shell; CA1, CA1 of hippocampus; CPu, caudate/putamen; DR, dorsal raphe, DRD, dorsal part; DRI, interfascicular part; DTT, dorsal tenia tecta; fmi, forceps minor of the corpus callosum; IOC, inferior olive, central part; IOD, inferior olive, dorsal part; IP, interpeduncular nucleus: IPC, caudal part; IPDL, dorsolateral part, IPI, intermediate part, IPL, lateral part, IPR, rostral part; ipf, interpeduncular fossa; LH, lateral hypothalamus; LM, lateral mammillary nucleus; LSI, lateral septal nucleus, intermediate part; mfb, medial forebrain bundle; MGP, medial globus pallidus; mlf, medial longitudinal fasciculus; MS, medial septum; MnR, median nucleus raphe; opt, optic tract; PH, posterior hypothalamus; PMnR, paramedian nucleus raphe; PnO, pontine reticular nucleus; PrL/IL, prelimbic/infralimbic cortex; Rbd, rhabdoid nucleus; RMTg, Rostromedial tegmental nucleus; ROb, raphe obscurus nucleus; RSA, retrosplenial agranular cortex; RSG, retrosplenial granular cortex; SuM, supramammillary nucleus; VDB, ventral diagonal band; VTA, ventral tegmental area; VTg, ventral tegmental nucleus; xscp, decussation of the superior cerebellar peduncle. Scale bars: 1 mm (all atlas views) and 200 μm (all fluorescence views).

**Figure 13.**
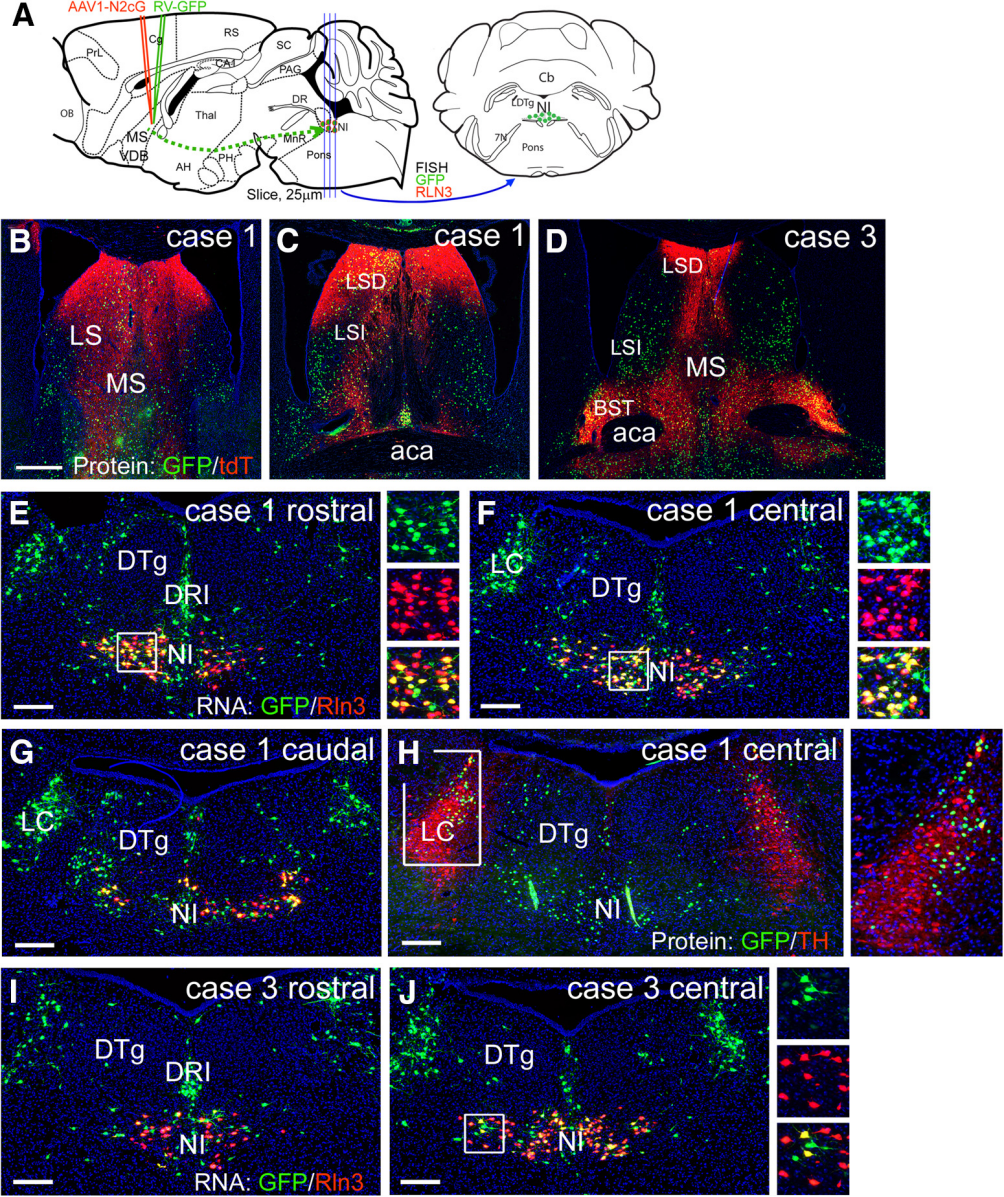
Transsynaptic tracing of the NI projection to GABAergic neurons in the septum. ***A***, Strategy for the transsynaptic labeling of the NI projection to GABAergic neurons in the septum (Materials and Methods). Results from two out of six injected cases are shown. ***B***, ***C***, Injected area from case 1, shown in sections corresponding to bregma 0.74 (***B***) and bregma 0.14 (***C***) in a standard atlas. ***D***, Injection case 3, corresponding to bregma 0.38. ***E–G***, Sections through the rostral, central, and caudal NI from case 1, showing the expression of endogenous Rln3 mRNA and RV-encoded GFP RNA using FISH. ***H***, A section from the central NI in case 1 adjacent to (***F***) showing expression of endogenous tyrosine hydroxylase immunoreactivity and virally-encoded GFP protein expression. ***I***, ***J***, Sections through the NI in case 3, which exhibited sparser retrograde labeling in a pattern similar to Case1. aca, anterior commissure; BST, bed nucleus stria terminalis; DRI, dorsal raphe, interfascicular part; DTg, dorsal tegmental nucleus; LC, locus coeruleus; LS, lateral septum; LSD, dorsal part; LSI, intermediate part; MS, medial septum; NI, nucleus incertus. Scale bars: 400 μm (***B***) and 200 μm (***E–J***).

The NI of Rln3^C^*^re^* mice was injected with a mixture of Cre-dependent AAV-expressing sypGFP and tdT as shown in Figure 7, strategy 2. The summary is based on the case appearing in Figures 10, 11, but further supporting data were derived from the case shown in Figures 12, 13, and injected cases not shown. The density of afferents from the NI was scored from + to +++ based on the appearance of punctate sypGFP fluorescence, which is consistent with presynaptic labeling. The designation +/− is reserved for areas in which tdT-labeled fibers were observed, but little or no sypGFP, suggesting labeled fibers of passage. No consistent Rln3^C^*^re^*-dependent labeling was observed in areas caudal to the pons.

1 Labeled fibers appear immediately adjacent to the IP, but little synaptic labeling is seen within the nucleus.

2 The rostromedial tegmental nucleus is not defined in standard atlases (Paxinos and Franklin, 2001) but resides just dorsolateral to the IP; this area is known to send GABAergic input to the VTA (Quina et al., 2015).

3 sypGFP labeling is defined within the VTA as anatomically defined, but it does not appear adjacent to DA neurons in this area.

4 The area defined as the anterior tegmental nucleus in standard atlases has been shown to contain neurons with similar properties to the RMTg, and has been labeled the caudal RMTg (cRMTg, Quina et al., 2015).

The NI is often identified by its characteristic Rln3 expression, yet this anatomic region contains both Rln3-expressing and Rln3-non-expressing neurons, and it is not clear whether these populations project to distinct targets. In order to distinguish the projections of Rln3-expressing and Rln3-non-expressing neurons in the NI, we employed a dual AAV strategy in which GFP expression is activated by *Rln3^Cre^*, and tdTomato expression is inactivated (FLEX-GFP/FAS-tdT, strategy 3; Fig. 7*F*). The cortex and septum appeared to be preferentially innervated by Rln3-expressing neurons (Fig. 12*A–F*). In contrast, Rln3-non-expressing neurons showed preferential innervation of the medial thalamus (Fig. 12*G*), the lateral mammillary nucleus (LM; Fig. 12*I*,*J*), and the IP (Fig. 12*M*,*N*). The VDB showed input from both sources (Fig. 12*D*), as did the LH and adjacent VTA (Fig. 12*H–L*), and the MnR/PMnR (Fig. 12*O*,*P*), whereas the rhabdoid nucleus (Rbd) showed input from Rln3-non-expressing neurons only. Dense fiber inputs to the central part, and only the central part, of the inferior olive (IO) were noted, exclusively from Rln3-non-expressing neurons (Fig. 12*Q*,*R*). These results indicate that the NI/Rln3 neurons give rise to a set of efferents that are often distinct from their immediate neighbors, and that a targeted genetic strategy is necessary to selectively label and manipulate the function of the NI-Rln3 pathway.

Prior work on NI function has focused on its input to the septohippocampal system (Nuñez et al., 2006; Ma et al., 2009b; Cervera-Ferri et al., 2012; Martínez-Bellver et al., 2015, 2017), and the NI has been suggested as a modulator of hippocampal theta rhythm. The presence of dense NI-Rln3 fiber endings in the septum suggest a pathway for the modulation of theta, in that MS GABAergic neurons project to CA1, where they synapse on other GABAergic neurons, which in turn modulate hippocampal pyramidal cell activity (Unal et al., 2015). Both enhancement and inhibition of theta activity by the manipulation of the NI have been reported (Szőnyi et al., 2019; Lu et al., 2020), but the relevant synaptic pathway from NI-Rln3 neurons to the septohippocampal system has not been determined.

In order to test whether NI-Rln3 neurons, and/or other cell types in the NI, have a direct synaptic connection to septal GABAergic neurons we employed RV transsynaptic tract-tracing using the modified RV EnvA CVS-N2cΔG-histone-eGFP (RV-GFP, Lo et al., 2019) and a tricistronic, cre-dependent helper AAV1, that expresses the pseudotyping receptor TVA, tdTomato, and the rabies glycoprotein G (AAV1-N2cG; Fig. 13*A*; Materials and Methods). AAV1 was activated by transgenic Cre-recombinase expression in *Gad2^Cre^* mice. AAV1-N2cG was injected on day 1 of the labeling protocol, followed by RV-GFP 21 d later, to allow for expression of the AAV-encoded RV proteins, followed by an additional 10 d to allow for retrograde labeling before harvesting the brain tissue. AAV-expressed tdTomato and RV-expressed nuclear GFP were observed in the septum and adjacent brain regions (Fig. 13*B–D*). One case, which heavily labeled the MS and LS, resulted in frequent labeling of both Rln3-positive and a smaller population of Rln3-negative cells in the NI (Fig. 13*E–G*); retrograde labeling was also observed in neurons in the inferior dorsal raphe (DRI, Fig. 13*E*) and in dorsal part of the locus coeruleus, identified by the expression of tyrosine hydroxylase (Fig. 13*H*). A second case is also shown in which the AAV/RV injection was placed more caudally, in the caudal part of the septal complex, extending into the BST, where few NI fibers terminate. This resulted in a sparser but qualitatively similar pattern of NI labeling, including both Rln3-positive and Rln3-negative NI neurons (Fig. 13*I*,*J*).

**Figure 14.**
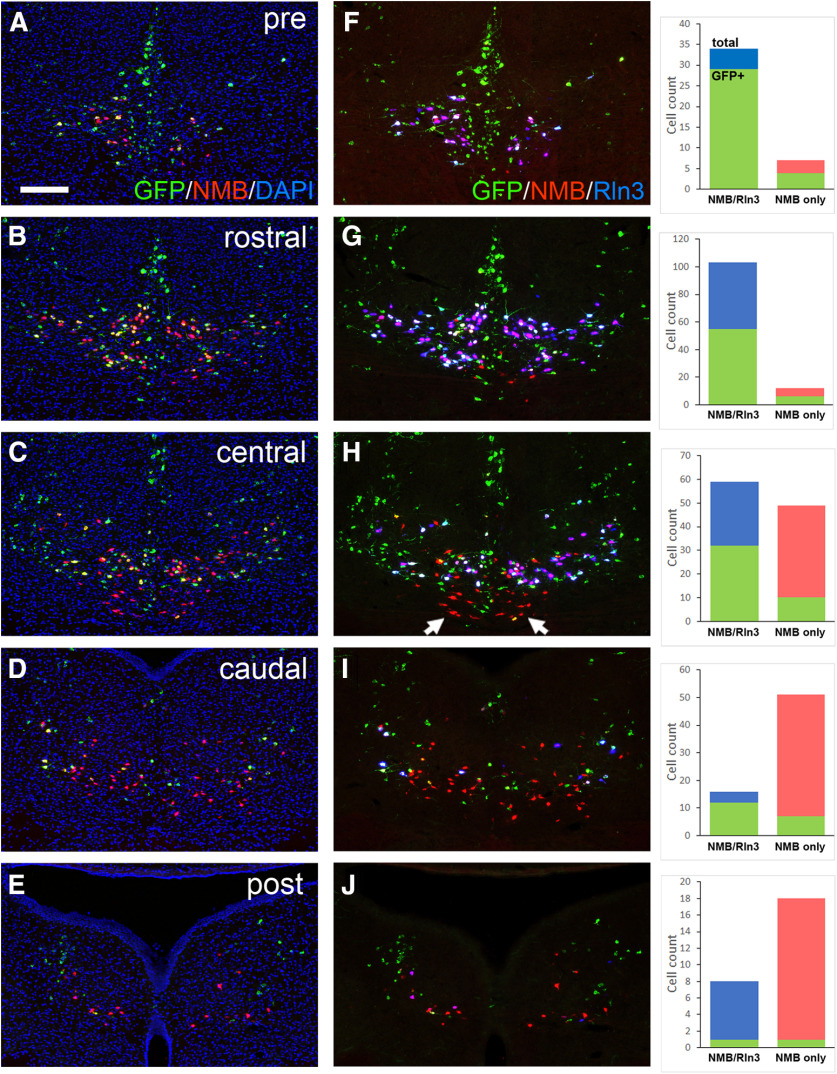
Transsynaptic retrograde labeling of NI Rln3/NMB and NMB-only neurons from the septum. Triple-label FISH for Rln3, NMB, and GFP mRNA was performed on a series of sections encompassing the NI and the areas immediately rostral (pre) and caudal (post) to the NI. Sections are from an extended series from the same injected case and adjacent to those shown in Figure 13*E–G*. Co-localization of mRNA signals was verified using confocal microscopy. GFP expression was assessed in Rln3/NMB and NMB-only neurons. Neurons expressing Rln3 alone were rarely observed. ***A–E***, FISH for GFP and NMB, together with DAPI nuclear staining to show the anatomic localization of the sections. ***F*–*J***, Images of the same sections shown in ***A–E*** with co-localization of Rln3, NMB, and GFP, and cell counts for the expression of GFP in the Rln3/NMB and NMB-only populations. NMB-only neurons are concentrated at the midline of the central NI (arrows, ***H***) and in the caudal part of the nucleus (***I***, ***J***), and are less likely at all levels to be labeled by RV-GFP from septal projections. Scale bar: 200 μm.

In order to better determine the relationship between the neuronal cell types in the NI region and the likelihood of retrograde RV-mediated labeling from the septum we used triple-label FISH to assess RV-GFP expression in Rln3+/NMB+ and Rln3–/NMB+ NI neurons (Fig. 14). Rln3+/NMB+ neurons were concentrated in the rostral and central NI (Fig. 14*A–C*,*F–H*), and in this area, 59% of Rln3+/NMB+ neurons were GFP-positive. Rln3–/NMB+ neurons were concentrated in the central to caudal NI (Fig. 14*C–E*,*H–J*), where 15% of Rln3–/NMB+ neurons expressed RV-GFP. In the central NI Rln3–/NMB+ neurons are found mainly near the midline (Fig. 14*H*, arrows). Rln3–/NMB+ neurons were less likely at all levels to be labeled by RV-GFP from septal projections, and in the most caudal sections, Rln3–/NMB+ neurons projecting to the septum were rare. These results support the conclusion that the Rln3+/NMB+ and Rln3–/NMB+ neurons are specialized with respect to both their anatomic distribution and their projections, and that the Rln3–/NMB+ neurons make much less of a contribution to the NI-septal pathway.

## Discussion

### Defining cell populations in the NI and adjacent pontine tegmentum

In the present study, we have used transgenic expression of Cre-recombinase in Rln3-expressing neurons to characterize the cellular phenotype and efferent projections of NI-Rln3 neurons, and also to define the specific projections of smaller populations of Rln3-expressing neurons in the PAG and DpMe. Expression of Rln3 has been used as a defining marker of the NI in rodents and primates (Burazin et al., 2002; Ma et al., 2007, 2009a), although the area encompassing the anatomic NI contains a mixture of Rln3-expressing and Rln3-non-expressing neurons (Ma et al., 2007). Recently, expression of Cre-recombinase targeted to the *Nmb* gene has also been used for functional studies of the NI (Lu et al., 2020). The present results help to clarify the neurotransmitter identity of neurons in this region of the pontine tegmentum as a basis for future functional studies. Lu et al., reported that ∼50% of NMB-expressing neurons in the NI also express Rln3, although only a single anatomic level was described (Lu et al., 2020). Here, in the region usually defined as the NI, we observe a higher degree of co-expression of NMB and Rln3 mRNA, with a minor population of neurons expressing NMB alone. However, at the caudal pole of the NI, NMB-positive, Rln3-negative neurons predominate, and neurons with this molecular signature extend caudally within the pontine CG into areas not usually defined as part of the NI, to the level of the PDTg and the appearance of the facial (seventh) nerve at the transition from the pons to the medulla. We conclude that Rln3 expression is specifically correlated with the area usually defined as the NI, and that NMB-expressing neurons encompass the NI plus additional neurons in the caudal pontine tegmentum that may or may not have similar functions.

NI-Rln3 neurons have been previously shown to express GABAergic markers (Ma et al., 2007). Here, we observed that both Rln3-expressing and NMB-expressing neurons within the NI consistently co-expressed mRNA for the GABAergic marker GAD2, and co-expression of mRNA for the glutamatergic marker VGluT2 was rarely if ever observed. Using a virally-introduced reporter for NMB-Cre, Lu et al. reported that 76% of the NI NMB-expressing neurons are GABAergic based on co-expression of the vesicular GABA transporter VGAT, but they did not examine co-expression with a glutamatergic marker. Given that imaging of mRNA is a more direct method of assessing these markers than Cre-mediated viral expression of a fluorescent protein, we conclude that both Rln3-expressing and NMB-expressing neurons within the NI and adjacent pontine tegmentum are all or nearly all GABAergic.

### Efferent pathways of Rln3-expressing neurons

The distribution of Rln3-expressing fibers in the mouse and rat brain has been previously examined by immunostaining (Ma et al., 2007; Smith et al., 2010), but this does not by itself reveal the source of the Rln3 immunoreactive fibers. Here, we have shown, using Cre-mediated viral tract-tracing, that the two relatively minor populations of Rln3-expressing neurons in the DpMe and PAG have restricted projections, and account for only a small part of the wide distribution of Rln3-immunoreactive fibers previously identified. Labeling of the DpMe cell group revealed very limited projections within the mesencephalon, close to the site of injection. Labeling of Rln3-expressing neurons in the midbrain PAG resulted in a few labeled fibers in the LH, PH, and ZI, but predominately labeled a tract running just inferior to the MG, and in the supraoptic decussation, which is known to connect the MG hemispheres. In contrast, injection of viral tracers into the NI of Rln3-Cre mice revealed extensive efferents, including projections to the hypothalamus, septum, hippocampus, and neocortical areas known from anatomic studies to receive extensive input from the NI (Olucha-Bordonau et al., 2003). Anterograde tracing results from *Rln3^Cre^* neurons in the DpMe, PAG, and NI described here, if taken together, recapitulate the entire set of Rln3 immunoreactive fiber terminals previously reported in the mouse (Smith et al., 2010).

Rln3-expressing neurons are a subset of the NMB-expressing neurons in the pontine tegmentum, and a comparison of the efferents of these neurons using *Rln3^Cre^* and *NMB^Cre^* mice suggests that Rln3+/NMB+ and Rln3–/NMB+ neurons may serve specific pathways. A prior anterograde tracing study in *NMB^Cre^* mice showed many projections in common with the Rln3-expressing neurons examined here, including efferents to the septohippocampal system, LH/LPO, and cortex (Lu et al., 2020). However, *NMB^Cre^* tract-tracing also prominently labeled pathways that are sparsely labeled or unlabeled using *Rln3^Cre^*, including the interpeduncular nucleus (IP, including its major rostral and caudal subdivisions IPR and IPC), the LM, and the IO. We observed strong fiber labeling in these areas only when the pontine tegmentum was injected with an AAV tracer that was not dependent on *Rln3^Cre^* (Fig. 12). Consistent with these results, Rln3 immunoreactive fibers are either absent (IP, excepting the dorsomedial part, IPDM), or sparse (LM, IO) in these areas (Smith et al., 2010). Since the main population of Rln3–/NMB+ neurons are found in a caudal region of the tegmentum that extends beyond the anatomic NI, the projections that are unique to *NMB^Cre^* mice are likely to arise from this area. These specific projections suggest distinct functions for the rostral Rln3+/NMB+ neurons and the caudal Rln3–/NMB+ neurons. The Rln3+/NMB+ neurons are the most likely candidates for interaction with the septohippocampal system and the regulation of hippocampal theta activity. Two areas that appear to receive input from Rln3–/NMB+ neurons, the LM and IO, are both implicated in motor function and motor learning, specifically head direction control (Clark and Taube, 2012) and cerebellar motor learning (Schweighofer et al., 2013), respectively. These neurons may be the best candidates for mediating the changes in locomotion noted with the optogenetic activation of NMB-expressing tegmental neurons (Lu et al., 2020), and are less likely to be mediated by the NI as usually defined. Specific, combinatorial genetic strategies will be needed to resolve the function of these pontine cell types.

Much of the prior work on NI function has focused on its input to the septohippocampal system and the regulation of hippocampal theta rhythm. Theta activity is observed in multiple regions of the extended hippocampal system, including CA1, CA3, and the DG, the MS/DBB, and the retrosplenial and entorhinal cortex, many of which receive NI-Rln3 inputs. In urethane-anesthetized rats, which exhibit slow (3–4 Hz) theta rhythms, electrical stimulation of the NI increases hippocampal theta (Nuñez et al., 2006). Some NI neurons are synchronized to induced (Martínez-Bellver et al., 2015) or spontaneous (Ma et al., 2013) hippocampal theta, and stimulation of the NI can shift the phase of theta (Martínez-Bellver et al., 2017).

The presence of dense NI-Rln3 fiber endings in the septum suggest a pathway for the modulation of theta. Many neurons in the MS fire at theta frequency *in vivo* (Borhegyi et al., 2004), and silencing the MS with nonspecific but reversible blockers eliminates theta activity in the hippocampus, and impairs spatial memory (Mizumori et al., 1989, 1990; McNaughton et al., 2006). In freely moving animals, the infusion of an Rln3 antagonist into the MS decreases theta power during arena exploration (Ma et al., 2009b). MS GABAergic neurons project to CA1, where they synapse on other GABAergic neurons, which in turn modulate hippocampal pyramidal cell activity (Unal et al., 2015). Here, using transsynaptic RV tracing, we have shown evidence for a direct link between NI-Rln3 neurons and septal GABAergic neurons that is a potential pathway for regulation of theta rhythms by the NI, although the sparser but widespread direct input of NI-Rln3 neurons to the hippocampus must also be considered.

The *Rln3^Cre^* mice used here, and the *NMB^Cre^* mice employed in a prior study (Lu et al., 2020), both allow for the labeling and manipulation of well-defined classes of GABAergic neurons in the tegmentum. However, another recent study of NI function used *Vgat^Cre^* transgenic mice to virally label “NI GABAergic neurons” as a single population (Szőnyi et al., 2019). Here, we have compared the restricted expression of Rln3 and NMB mRNA to the widespread expression of GABAergic markers in the tegmentum (Fig. 1*I-K*), and these results suggest that it is not feasible to specifically target the NI using Cre expressed in all GABAergic neurons. Consistent with this, reporter expression following viral injection of the pontine tegmentum in *Vgat^Cre^* mice was not restricted to the NI. Thus, some caution is warranted in attributing the physiological and behavioral results obtained using *Vgat^Cre^*-driven optogenetic stimulation specifically to the NI. This study reported an inhibition of hippocampal theta power on NI stimulation, while other studies, each with its own limitations, have reported enhancement of theta activity (Nuñez et al., 2006; Martínez-Bellver et al., 2017; Lu et al., 2020). A clear understanding of the relationship of NI activity and hippocampal theta rhythm will require the application of more specific tools that access specific cell types in the tegmentum with inputs to this system.

## Synthesis

Reviewing Editor: Karen Szumlinski, University of California at Santa Barbara Decisions are customarily a result of the Reviewing Editor and the peer reviewers coming together and discussing their recommendations until a consensus is reached. When revisions are invited, a fact-based synthesis statement explaining their decision and outlining what is needed to prepare a revision will be listed below. The following reviewer(s) agreed to reveal their identity: Thomas Jhou, David Root.

Synthesis:

Following consultation with two expert reviewers, we have come to the decision of “Revise and Re-review”. This decision was based on the many strengths of this report, to include the significant advancement in the ability to study Rin3 cells within brainstem using this novel transgenic system. However, our enthusiasm was dampened somewhat by shared concerns regarding the lack of quantification of immunostaining throughout the report, as well as issues related to the depiction of co-expression. In addition, the reviewers each raised some individual concerns that we hope will assist the authors in their revision. Overall, the issues raised are readily addressable and should not take longer than 2 months to complete. However, we understand if Covid-related restrictions to research materials/data might delay submission of the revision and if that is the case, please do not hesitate to contact eNeuro for an extension. Please find below the individual Reviewer comments:

Reviewer:

This excellent manuscript describes the creation and characterization of a Relaxin3-cre transgenic mouse. Using several strategies to visualize Cre-expressing neurons, and their efferents, along with dual-label RNAscope, the authors showed that the Rln3 cells are a subset of the GABAergic neuromedin B (NMB) cells in the NI, and that they project to a circumscribed set of targets in the forebrain, hypothalamus, and midbrain.

This is a well-documented and comprehensive study. Both the anatomical findings, and the transgenic mouse line, are valuable contributions to the field. The authors also do an excellent job of describing their findings in the context of prior work, including studies using NMB-Cre mice.

I have only a few comments, which should be easily addressable:

Reviewer 1

1. The manuscript notes that the Rln3-cre mice were made by inserting a Cre-GFP fusion protein downstream from the Rln3 stop codon. This would presumably introduce GFP into all Rln3-expressing neurons. Curiously however, there is no subsequent mention of this fluorescent label being present, and several other methods (cross with ZsGreen reporter, or virus injection) are instead used to visualize the Cre-containing neurons. Some clarification is needed here. Is this GFP fusion protein not expressed, or was it excised somehow? Or is it expressed only weakly? If the latter, how do the authors distinguish it from the other fluorescent labels of the same color?

2. I appreciate the atlas drawings placed alongside many of the high magnification photos. However, in figure 3, showing Rln3/NMB colocalization, it would have been nice to also have drawings of the overall section, or atlas diagrams, to show the exact rostro-caudal level at which the NMB-only neurons become predominant. For example, it would be good to see the Rln3 and NMB distributions in relation to other landmarks, such as the inferior colliculus. The existing high-power photographs do not allow this to be seen.

3. Figure 12. The insets in 12A are marked “C” and “D”, but appear to correspond to panels “B” and “D”. Panel “C” appears to have been skipped entirely.

4. Figure 13, figure legend, line 797, “asa” should be “aca”.

Reviewer 2

The nucleus incertus Rln3 system is not entirely characterized. The authors generated a Rln3 transgenic cre line and used multiple techniques to validate the line as well as identify the neuronal networks of these neurons. Cre and Rln3 showed exact correspondence and all were GAD-expressing while a subset were NMB-expressing. Homozygous mice showed reduced rln3 protein and heterozygous mice were used. DpMe projections were local, PAG projected to hypothalamus and near medial geniculate, NI projections reached cortical, hippocampal, hypothalamic, septal, and other sites. Most regions showed synaptophysin-GFP in these sites, suggesting vesicular release sites. NI rln3+ and rln3- projections often intermingled but had some spatial differences. Rabies tracing from septal GAD+ neurons showed NI rln3 innervation.

Major concerns:

1. My main concern of the manuscript is that much is not quantified throughout. Tables of sparse/strong labeling would be useful for projections. Counting cell bodies would give some useful information regarding how many of these different neurons exist.

2. Criterion for co-expression could use better explanation. This is related to the cellular overlap with vglut2. Can the authors decide whether “cellular overlap” explains vglut2 “co-expression”? It is possible with 25 um sections but if the authors believe this is the case this should not be counted as a co-expressing neuron. Alternatively if there are cells that co-express vglut2, the authors should not suggest cellular overlap. If the authors cannot be sure, thinner sections would be helpful.

3. For rabies starter neurons co-expressing tdT and GFP were the number of inputs related to starters?

4. fig7 strategy 3 - were any neurons TdT + GFP co-expressing?

5. Including more schematics in figures of strategies would be useful (e.g., fig 6, fig 7 strategy 3)

Minor concerns:

1. error on line 713. S, T should be switched

2. line 272 typo, period/comma

3. fig 12 missing c

4. Some discussion of whether rln3 and/or NMB being released by these neurons or only GABA, might be useful.
